# Evidence for a sensorimotor prediction in action observation

**DOI:** 10.1016/j.isci.2026.115876

**Published:** 2026-04-24

**Authors:** Pauline M. Hilt, Nicolas Gueugneau, Laurent Demougeot, Luciano Fadiga, Thierry Pozzo, Charalambos Papaxanthis

**Affiliations:** 1 Université Bourgogne Europe, CHU Dijon Bourgogne, INSERM, CAPS UMR 1093, 21000 Dijon, France; 2 Laboratory C3S, Culture Sport Health and Society, Sport and Performance Department, Université Marie et Louis Pasteur, Besançon, France; 3 Université Bourgogne Europe, CHU Dijon Bourgogne, Direction de la Recherche et de l’Innovation, 21000 Dijon, France; 4 Center for Translational Neurophysiology of Speech and Communication (CTNSC), Istituto Italiano di Tecnologia (IIT), Ferrara, Italy; 5 Department of Neuroscience and Rehabilitation, Section of Physiology, University of Ferrara, Ferrara, Italy; 6 GIS Starter, Dijon, France

**Keywords:** action observation, predictive coding, direct matching, motor prediction, motor imagery, locomotion, proprioceptive illusion, muscle vibration, mirror neuron system

## Abstract

The mirror neuron network is activated during both execution and observation of the same action, enabling visual-to-motor transformation. Two main hypotheses account for this process: the *direct matching* hypothesis proposes a direct link between visual cues and the observer’s motor representations, whereas the *predictive* hypothesis suggests that motor predictions are generated and compared with visual information. We tested these hypotheses during a locomotion task. Motor prediction was assessed by comparing actual and imagined walking (forward or backward) over a 9 m path. Action observation (AO) was examined through a time-estimation inference task in which participants observed an agent walking 3 m before occlusion and estimated when the agent would reach the end of the 9 m path. We manipulated these tasks by mechanically vibrating the leg muscles, creating sensory illusions. We found that vibration-induced sensory illusions altered motor imagery timing, affecting sensorimotor prediction. Opposite effects on AO and numerical simulations supported the *predictive* hypothesis.

## Highlights

• Sensory illusions modulate imagined, executed, and observed locomotion
• Motor imagery and action observation show opposite temporal biases
• Observation involves comparing predictions with visual input
• Findings support the involvement of predictive mechanisms in action observation

## Graphical abstract

**Figure:**
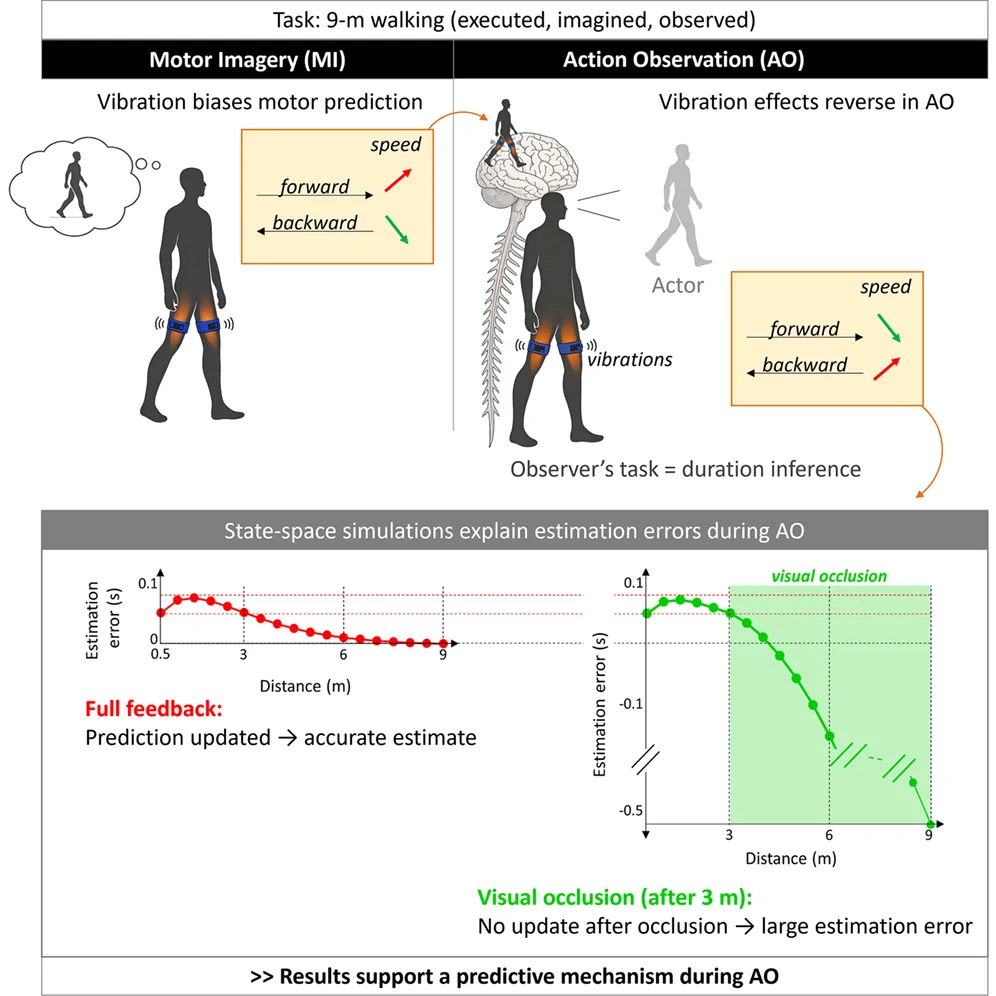

## Teaser

Neuroscience; Behavioral neuroscience; Cognitive neuroscience

## Introduction

Observing Olympic gymnasts perform complex and elegant movement combinations is always a fascinating and pleasant experience. In daily life, the ability to perceive and understand others’ actions is crucial for social communication and learning.^1^ Remarkably, in the human brain, a similar neural network is activated both during the observation and execution of the same action by the observer. This neural network, known as the “*mirror neurons network”* due to its particular properties, includes several brain regions, such as the motor,^2^^,^^3^ parietal,^4^^,^^5^^,^^6^ and prefrontal cortices,^7^ as well as the basal ganglia and cerebellum.^8^ Mirror neurons were first discovered in area F5 of the monkey premotor cortex.^9^^,^^10^^,^^11^ The presence of this neural network, which is similarly activated during both action execution and observation, may underpin a crucial process that enables humans to convert visual movement information into motor representations and to naturally and automatically understand the actions and intentions of others.^11^^,^^12^^,^^13^^,^^14^^,^^15^

The exact mechanism behind this transformation remains a topic of debate. To date, two main explanations have been proposed in the literature. The first, known as the *direct matching* hypothesis (see Figure 1A), suggests that mirror neurons map the observed action onto motor representations—automatic motor resonance of the frontoparietal areas.^14^ According to this hypothesis, there is a direct correspondence between the visual information extracted from the observed movement and the neural activations in the observer’s motor cortex that would produce the same movement. In other words, there is a direct link between visual input and motor output. In support of this view, neurophysiological evidence suggests that corticospinal excitability in the observer’s motor system faithfully replicates the spatiotemporal sequence of the observed action.^16^^,^^17^^,^^18^ For instance, observing index and little finger abductions selectively increases the corticospinal excitability of the first dorsal interosseous and abductor digiti minimi muscles, which control these finger movements, respectively.^19^

**Figure 1:**
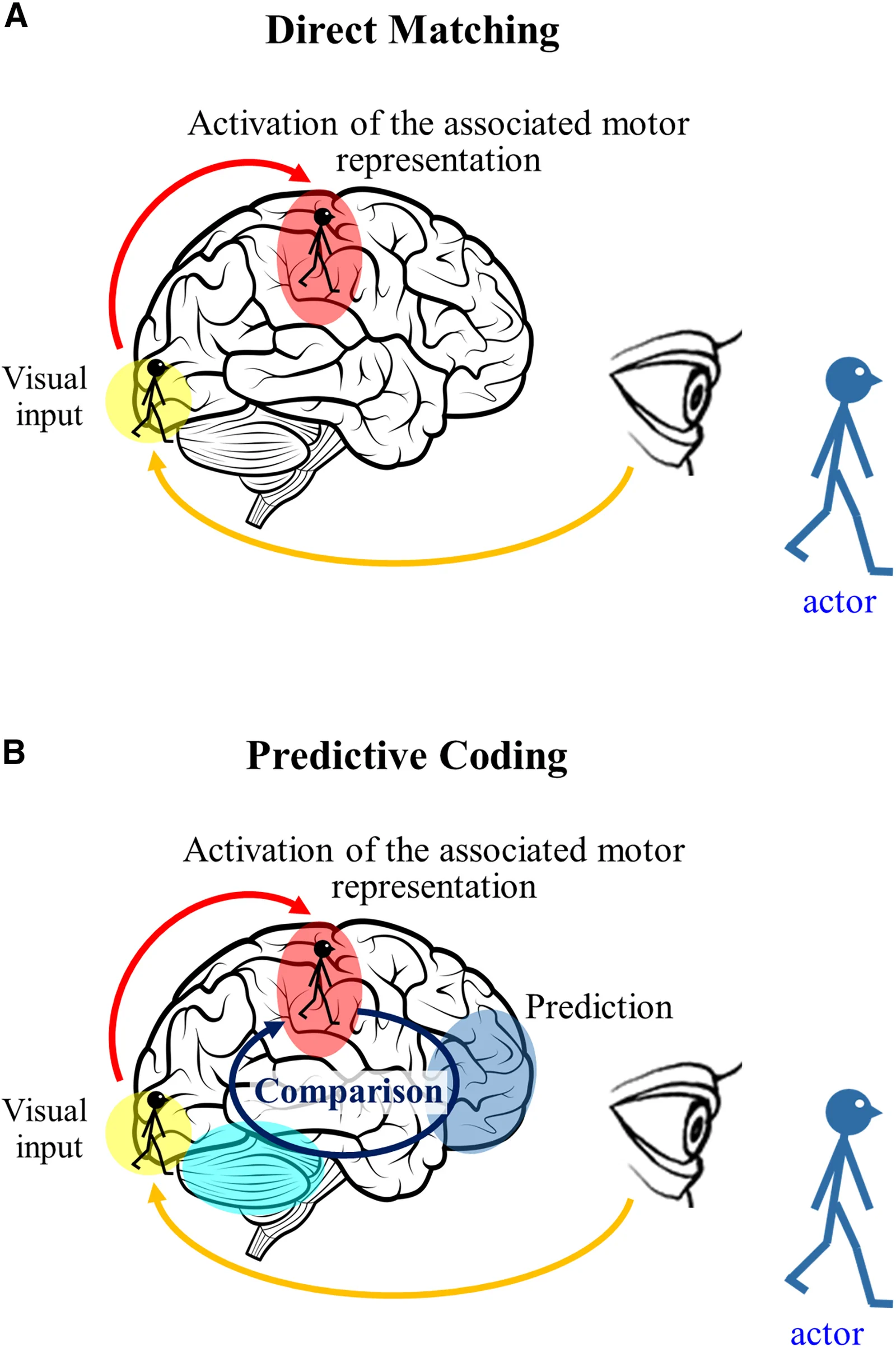
Illustration of the working hypotheses underlying action observation processes The figure provides an example of an observer estimating the walking speed of another person (agent) based on two competing theories. (A) Direct matching hypothesis: According to this hypothesis, visual information, extracted from observing walking movement directly, automatically activates the same motor representations in the observer’s motor cortex that would be involved in performing the same action. This process, which facilitates the estimation of the actor’s speed, is independent of the observer’s own motor signature (e.g., their unique walking pattern) or their current state, such as their posture during observation. (B) Predictive hypothesis: In contrast, the predictive hypothesis suggests that visual information from the observed walking movement activates the observer’s own motor signature—the specific motor pattern they would use if they were in the actor’s position. An internal forward model then uses a copy of these motor patterns to generate state predictions, such as the actor’s walking speed. This prediction is continuously updated through a comparison process with incoming visual information, gradually refining the estimate of the actor’s speed. Since the internal forward model incorporates both the motor command and the observer’s current sensorimotor state, the observer’s estimate of the actor’s speed could be influenced by their own current state.

The direct matching hypothesis is compelling due to its simplicity and may be effective for simple mono-articular movements.^20^ However, its applicability to more complex movements is questionable, mainly due to the well-known inverse problem. There is no unique solution that allows one to deduce from movement kinematics (i.e., the visual information of the observed movement), the appropriate motor commands (producing the same movement). In multi-joint movements, the abundance of degrees of freedom allows for multiple joint configurations and spatiotemporal patterns of muscle activity to achieve the same movement.^21^ Moreover, the *direct matching* hypothesis cannot convincingly explain how observers could learn or improve their motor repertoire by observing novel actions for which their motor representations have not yet been consolidated. Nevertheless, there is extensive literature on the effectiveness of action observation in motor learning (for a review,^1^^,^^22^).

Alternatively, the *predictive* hypothesis (see Figure 1B), based on the concept of motor prediction, could provide a solution to the inverse problem during action observation. In theory, during movement execution, sensorimotor predictions are generated by internal forward models—neural networks that simulate the causal flow of physical processes by predicting future sensorimotor states (e.g., position, velocity) based on an efferent copy of the motor command and the current state.^23^ A self-supervised learning process minimizes prediction errors, namely the errors between the output from the forward model and the sensory outcome (sensory feedback) of the motor command. This process is fundamental to sensorimotor learning and adaptation.^24^

Two interesting theories in cognitive neuroscience propose motor prediction as a fundamental process in the brain: active inference^25^ and internal model.^26^ Here, we focus on the motor control theory of motor prediction. According to this theory, during action observation, the observer’s motor system generates predictions about the likely sensory consequences of the observed movement. These predictions are informed by the observer’s current state, including proprioceptive and vestibular inputs, and are processed by forward models, which are thought to rely heavily on cerebellar computations. The forward model anticipates kinematic features of the observed action—such as speed and direction—based on prior experience and the structure of the movement itself. These predictions are continuously compared with incoming visual information. When discrepancies arise between expected and actual sensory input, a prediction error is generated, which can influence how the movement is perceived and interpreted. The involvement of the cerebellum and basal ganglia during action observation supports the existence of such comparison mechanisms.^8^ This scheme offers a biologically grounded explanation of how perceptual judgments about others’ actions can be shaped by internal prediction and error-monitoring processes.

Although the *predictive* hypothesis is more complex than the *direct matching* one, because it involves additional operations and neural circuits, it offers a unique advantage by proposing a forward process from motor commands to kinematics. While theoretical studies support the *predictive* hypothesis,^25^^,^^27^^,^^28^^,^^29^ empirical evidence remains limited. Interestingly, neurophysiological studies have suggested that the motor system computes the difference between the expected and observed actions. For example, a recent TMS study showed that during the observation of complex multi-joint actions, the primary motor cortex shows greater activation to differences than to similarities between perceived movements and the observer’s motor patterns.^30^ Additionally, cumulative evidence highlights the involvement of the cerebellum during action observation, a structure whose role in motor prediction is well established.^8^^,^^31^^,^^32^

In our study, we aimed to distinguish between the *direct matching* and the *predictive* hypotheses in action observation. We employed a locomotor task where participants (observers) observed an agent walking forward or backward and were asked to estimate when the agent would reach the end of a 9-meter path. Observers had a complete vision of the first 3 m of the walk, after which their view was occluded for the remaining 6 m. The primary measure was the temporal estimation error, that is, the difference between the actual time taken by the agent and the duration estimated by the observers. To assess motor prediction, we used the motor imagery paradigm.^33^^,^^34^ Forward models are supposed to be involved in imagined movements,^23^^,^^35^^,^^36^^,^^37^ and their temporal features are thought to emerge from sensorimotor predictions.^38^^,^^39^ In our protocol, participants first imagined walking the 9-meter path (forward or backward) and then physically performed it. The temporal differences between imagined and actual movements served as the key measure of motor prediction accuracy.^33^^,^^34^^,^^38^^,^^39^ By comparing temporal errors across the observation and imagined locomotion tasks, we sought to gain insights into the prediction processes underlying action observation and imagery. Notably, during imagined movement, motor prediction could not be updated by any sensory feedback—i.e., the predictions generated by the forward model remain untested and are used directly to estimate movement parameters such as duration, while action observation could benefit from visual input and thus recurrently adjust predictions—i.e., the generated predictions can be compared with actual sensory input, enabling the detection of prediction errors.

To challenge the three previous tasks (observation, imagined, and actual walking), we induced sensory illusions through mechanical vibrations of the leg muscles.^40^ These vibrations are known to produce directionally specific proprioceptive illusions of motion, thereby biasing the expected sensory consequences of movement. Importantly, they also create persistent directional effects on movement timing during both actual^40^ and imagined locomotion,^41^^,^^42^^,^^43^ directly influencing the sensorimotor predictions in these modalities. According to the *predictive* framework, action observation involves generating sensory predictions via internal forward models. These models simulate the dynamics of the observed action (e.g., its speed and direction) based on motor representations activated in the observer. Predictions rely on a combination of the motor command (or its internal simulation) and the current estimate of the body state. By altering proprioceptive signals, muscle vibration distorts this internal estimate of the body state, thereby biasing the predicted sensory consequences of movement. If action observation engages predictive mechanisms, then such biased predictions should be compared to incoming visual information, resulting in directionally specific prediction errors. This, in turn, should lead to systematic changes in the perception or inference of the agent’s movement—particularly in its estimated duration—reflecting modifications in prediction error signals. Therefore, we hypothesized that observers’ temporal estimation of the agent’s movement would be systematically shifted by the vibration’s effect, thereby revealing the operation of internal forward models during action observation. Conversely, if no specific effect of vibration were observed, it would suggest that sensorimotor prediction and comparison processes, well established during action execution and imagery, are not engaged during the observation of others’ actions—thus challenging the core assumption of prediction (see Figure 1B).

## Results

In the Experiments presented hereafter, participants were asked to observe, imagine, and walk along a 9-meter linear path. Additionally, we applied bilateral vibration to the tibialis anterior and hamstring muscles during the three tasks (see Figure 2). None of the participants had previous experience with vibratory stimulation. Yet, they all demonstrated sensitivity to it, exhibiting both illusory perceptions and postural reactions consistent with those reported in the literature.^40^ Specifically, tibialis anterior vibration (85 Hz) induced a forward trunk inclination (6.9° ± 2.6°) accompanied by an illusory backward tilt. In contrast, hamstring vibration produced a backward trunk inclination (−7.3° ± 3.8°), a forward shank inclination (9.4° ± 3.5°), and an illusory forward leg flexion (Ivanenko et al., 2000). These effects were reported consistently by all participants.

**Figure 2:**
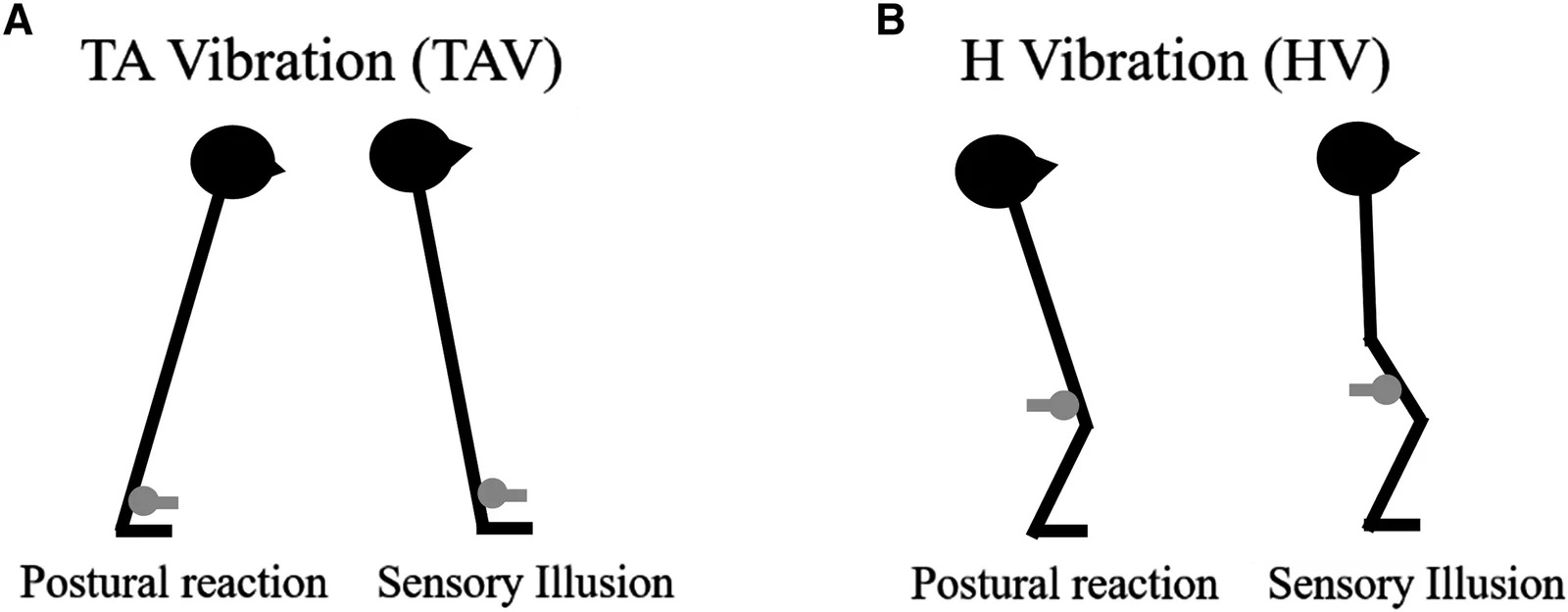
Muscle vibration The tibialis anterior (A) and hamstring (B) muscles were bilaterally stimulated by two mechanical vibrators at 85 Hz. The vibrators were fixed on the tibialis anterior (∼4 cm above the ankle joint) and the hamstring distal (∼3 cm above the knee joint) tendons. Qualitative illustrations of the postural and sensory illusions caused by muscle vibration are depicted. Tibialis anterior muscle vibration (A) induced a forward body tilt and an illusory perception of backward body tilt. Hamstring muscle vibration induced a backward inclination of the trunk and a forward inclination of the shank, and an illusory forward leg flexion relative to the trunk (B).

### Experiment 1: Imagined locomotion

In the first Experiment (Figure 3A), we investigated how muscle vibration affects motor prediction, which was measured by the duration of imagined locomotion (mental chronometry paradigm). This Experiment focused exclusively on imagined movements to ensure that participants had no prior experience of the effects of muscle vibration on actual movement. Nine volunteers imagined walking forward or backward along a 9-m path at a natural speed, under three conditions: no vibration (NoV), tibialis anterior vibration (TAV), and hamstring vibration (HV). Each condition included 12 trials. Participants indicated the imagined start and end using a stopwatch. They reported vivid kinesthetic sensations in all conditions (mean = 5.53 ± 0.96 on a 7-point Likert scale).

**Figure 3:**
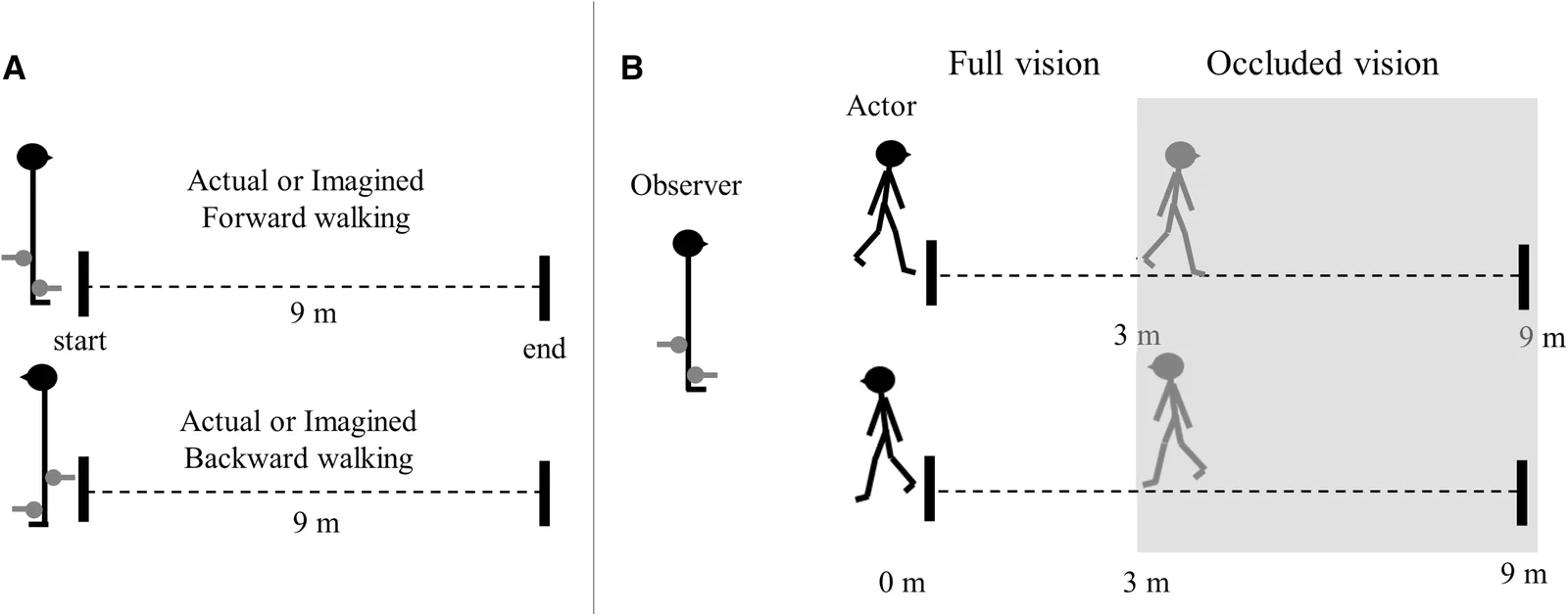
Experimental procedures (A) Imagined and actual locomotion. Participants mentally (Experiment 1) and physically (Experiment 2) walked forward and backward along a 9m linear path at a natural speed with complete vision and with or without vibration of the tibialis anterior and hamstring muscles. (B) Locomotion inference (Experiment 3). The participant (observer) watched a human actor walking along the 9m path (forward, top; backward, bottom) at a natural speed with or without vibration of the tibialis anterior and hamstring muscles. When the actor reached 1/3 of the path (3m), the observer’s vision was occluded by crystal goggles until the end of the trial. The observer thus had to estimate when the actor would reach the end of the path.

The average duration (±SD) of imagined locomotion is shown in Figure 4A. The ANOVA revealed a significant interaction between *vibration* and *direction* (F(2,16) = 112.6, *p* < 0.0001, *η*^2^ = 0.94). To clarify this interaction, post-hoc analyses showed that imagined forward locomotion was consistently faster than backward locomotion within each vibration condition (all *p* < 0.001). Vibration of the tibialis anterior muscle did not modulate imagined locomotion (*p* > 0.1 for both directions). In contrast, hamstring vibration accelerated imagined forward locomotion (*p* < 0.001; on average −8.25%, range −4.6% to −13.1%) and slowed imagined backward locomotion (*p* < 0.001; on average + 7.59%, range +5.7% to +9.8%). Imagined durations remained stable across repetitions (Figure S1A): comparing the first three and last three trials revealed no significant differences (all t < 1, *p* > 0.1).

**Figure 4:**
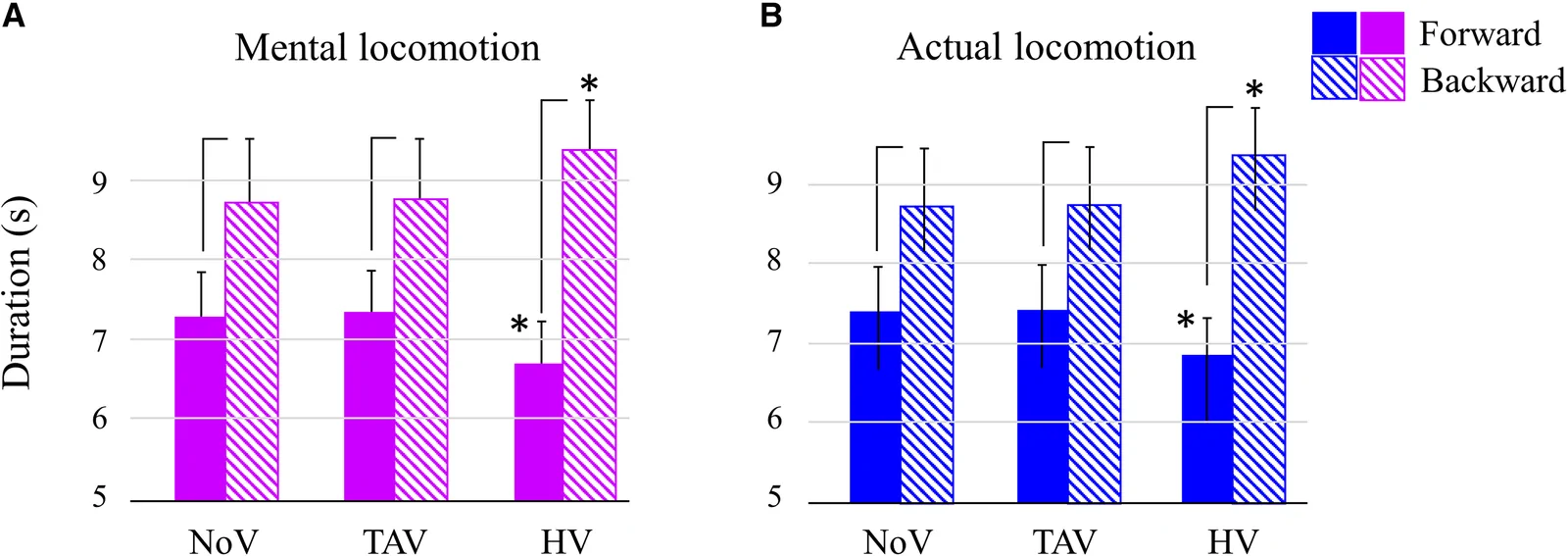
Effects of lower-limb muscle vibration on the duration of imagined and executed forward and backward locomotion Mean durations (+SD) of forward (full bar) and backward (striped bar) locomotion in the mental (A, Experiment 1) and actual (B, Experiment 2) locomotor tasks for each vibration condition. Black lines indicate significant differences between the walking directions (forward versus backward). Stars indicate significant differences between the HV condition and the two other vibration conditions (NoV and TAV) within each direction of locomotion (forward and backward). NoV, no vibration; TAV, tibialis anterior muscle vibration; HV, hamstring muscle vibration.

Vibration effects on imagined locomotion depend on the stimulation frequency and, consequently, on the illusory effects it evokes. In control Experiment 1a (see Table S1), where five participants from Experiment 1 followed the same protocol, the vibration of the hamstring muscle at 40 Hz (this frequency does not induce any sensory illusion or postural effects) did not alter forward (permutation test: *t* = 0.03, *p* = 0.96, *d* = 0.01) or backward (permutation test: *t* = 0.04, *p* = 0.98, *d* = 0.01) imagined walking speeds.

Additionally, the main findings of Experiment 1 could not be attributed to postural effects elicited by muscle vibration (see Figure 2). To confirm this, we asked five participants from Experiment 1 to repeat the mental locomotion task (only the forward condition and without vibration), voluntarily adopting the postures induced by vibrating the tibialis anterior and hamstring muscles (control Experiment 1b; see Table S1). The results showed that, regardless of the participants’ initial position, the imagined walking speed remained the same (TAV-posture vs. natural erect posture: permutation test; *t* = −0.12, *p* = 0.86, *d* = 0.04; H-posture vs. natural erect posture: permutation test; *t* = 0.04, *p* = 0.96, *d* = 0.01).

### Experiment 2: Actual locomotion

In the first Experiment, we found that hamstring muscle vibrations specifically influenced the duration of imagined locomotion. In the second Experiment, conducted at least one week later, we investigated whether similar effects would be observed during actual locomotion. The same participants were asked to walk at a natural pace, with full vision, either forward or backward along the 9-meter linear path, under the same three experimental conditions (Figure 3). Each participant completed 12 trials per condition, and the walking duration was recorded using an electronic stopwatch. The participants activated the stopwatch when they began walking and stopped it upon reaching the end line.

The average walking duration (±SD) is shown in Figure 4B. The ANOVA revealed a significant interaction between *vibration* and *direction* (F(2,16) = 104.1, *p* < 0.0001, *η*^2^ = 0.93). Post-hoc analyses showed that forward walking was faster than backward walking within every vibration condition (all *p* < 0.001). As in the imagery task, tibialis anterior vibration did not affect walking speed (*p* > 0.1 for both directions). Hamstring vibration accelerated forward walking (*p* < 0.001; average −8.72%, range −5.2% to −13.5%) and slowed backward walking (*p* < 0.001; average + 7.71%, range +6.3% to +9.9%). Durations were highly consistent across repetitions (Figure S1B), with no significant change between the first and last trials (all t < 1, *p* > 0.1). These findings corroborate those of previous studies that reported the specific effects of leg muscle vibration on walking speed.^40^

We also confirmed with five previous participants (control Experiment 2) that vibration of the hamstring muscle at 40 Hz did not alter forward (permutation test; *t* = 0.07, *p* = 0.91, *d* = 0.02) or backward (permutation test; *t* = 0.11, *p* = 0.85, *d* = 0.04) walking speeds (see Table S1).

### Experiments 1–2: Comparison of imagined and actual locomotion

The vibration of the hamstring muscle produced comparable effects on both actual and imagined locomotion duration. Note that these effects were present in all participants, without exception. The statistical comparison of the vibratory effects, assessed by the ratio $\left[\frac{HV-NoV}{NoV}\times 100\right]$ and calculated for actual and imagined locomotion separately, did not show any impact on forward (*t* = 2.21, *p* = 0.12, *d* = 0.74) or backward locomotion (*t* = −0.62, *p* = 1, *d* = −0.21; Figure 5A). Notably, we found strong correlations between the actual and imagined durations under hamstring muscle vibration for forward (*r* = 0.97, *p* < 0.0001, *t* = 11.26; Figure 5B) and backward (*r* = 0.92, *p* < 0.0001, *t* = 6.35; Figure 5C) locomotion; the greater the effect of hamstring muscle vibration on actual locomotion, the greater its effects on imagined locomotion.

**Figure 5:**
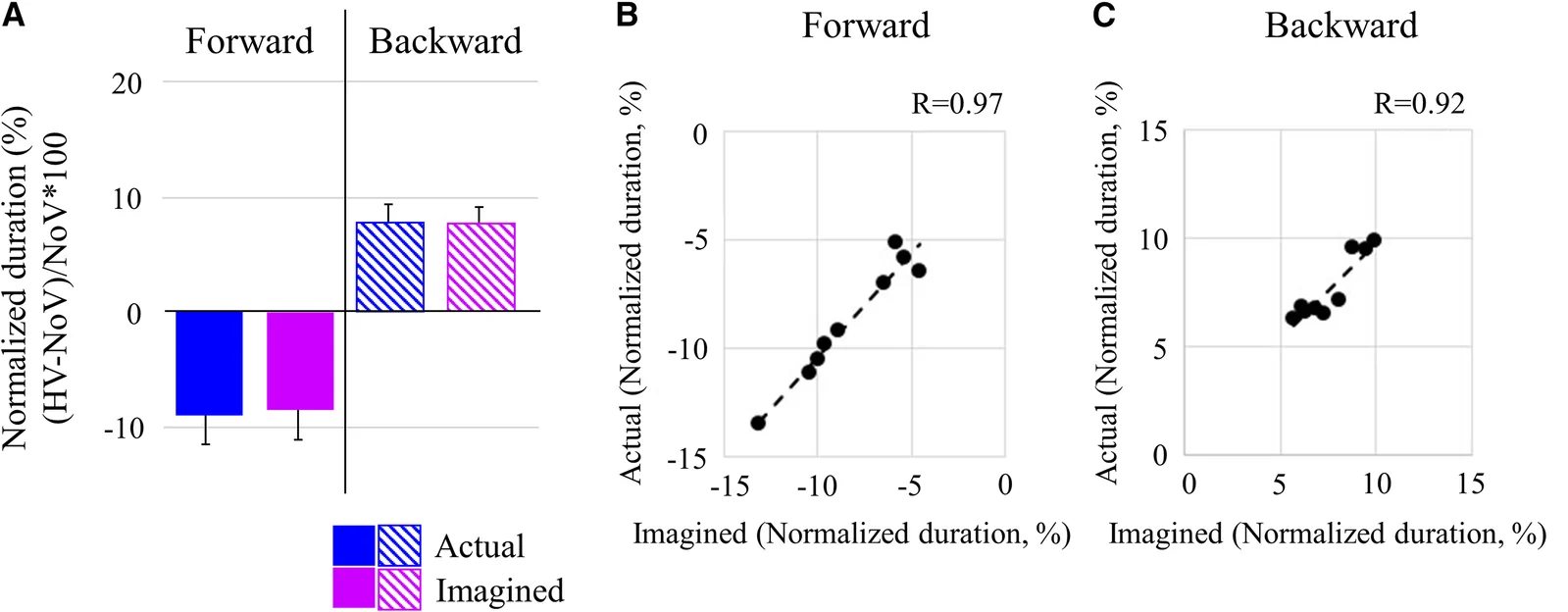
Comparison and correlation of hamstring vibration effects on actual and imagined locomotion (A) Effects of hamstring muscle vibration (normalized against no vibration, NoV) on actual and imagined locomotion for forward (full bar) and backward (striped bar) walking. Correlations between actual and imagined durations under hamstring muscle vibration for forward (B; *r* = 0.97, *p* < 0.0001, *t* = 11.3) and backward (C; *r* = 0.92, *p* < 0.0001, *t* = 6.35) locomotion; the greater the effect of hamstring muscle vibration on actual locomotion, the greater its effects on imagined locomotion.

### Experiment 3: Locomotion observation

In the previous experiments, we demonstrated that hamstring muscle vibrations directly influence the timing of imagined walking movements, suggesting that motor imagery relies on a sensory prediction process.^41^^,^^42^ These effects were also observed during actual locomotion, ruling out the possibility that muscle vibration merely acts as an additional load that disrupts locomotion speed. The fact that vibration at 40 Hz does not produce sensory or postural effects further supports this conclusion.

In the third Experiment, we explored the effects of muscle vibration on action observation. We therefore recruited a new group of nine volunteers for a locomotion observation task. Each participant (referred to as “observer”) was asked to estimate the time it would take another individual (referred to as “agent”) to walk the same 9-meter linear path (Figure 3B). The agent walked forward or backward with full vision at the observer’s natural walking speed. The observer was able to fully observe the agent’s locomotion for the first 3 m of the path. For the remaining 6 m, the observer’s vision was occluded by crystal goggles equipped with a wireless obstruction system (Plato System, 2 ms resolution). The actual walking duration and the corresponding time estimated by the observer were recorded using two electronic chronometers (manipulated by the agent and the observer, respectively), synchronized through a computer program (see STAR Methods section for details). The observer completed the task (12 trials per condition) under the same experimental conditions (NoV, TAV, and HV; Figure 3) as in the previous experiments.

Based on the findings of the above experiments and the theoretical assumptions illustrated in Figure 1, we formulated specific hypotheses for Experiment 3. According to the predictive framework, action observation engages internal forward models that generate sensorimotor predictions based on prior motor experience and current sensory inputs. Muscle vibration alters proprioceptive input, thereby shifting the internal state from which predictions are computed. During imagined locomotion, this directly causes a change in the predicted movement pattern (e.g., faster simulated speed when hamstrings are vibrated during forward walking). During action observation, however, these biased internal predictions are compared with incoming visual information about the agent’s actual movement. If a mismatch arises—i.e., a prediction error—the observer adjusts their estimation accordingly.

Specifically, we hypothesized that hamstring vibration would produce a directional bias in speed prediction: accelerating internally simulated forward locomotion and decelerating internally simulated backward locomotion. During observation, this mismatch would result in an overestimation of the agent’s forward walking time (because the observed speed seems slower than predicted) and an underestimation of the backward walking time (because it seems faster than predicted). This reversal relative to the effects observed in motor imagery is a key prediction of the predictive account.

In contrast, the direct matching hypothesis posits that observation activates motor representations corresponding to the observed action without any internal comparison. In this view, muscle vibration should not affect speed estimation, as the observers merely mirror the agent’s movement without considering their own altered sensorimotor predictions. At most, a nonspecific effect (e.g., attentional load) might slightly bias performance, but no direction-specific impact would be expected.

ANOVA applied to the observers’ temporal estimation (Figure 6: dark green bars represent the observer’s estimation and light green bars represent the actor’s duration), revealed an interaction effect between *vibration* and *direction* (F_(2.16)_ = 147.1, *p* < 0.0001, *η*^2^ = 0.95). While vibration of the tibialis anterior muscle did not affect temporal estimation (*p* > 0.1, for both forward and backward locomotion), hamstring muscle vibration induced an overestimation of the agent’s forward walking (*p* < 0.001; on average 10.26%; individual values ranged from 2.5% to 16.1%) and an underestimation of the agent’s backward walking (*p* < 0.001; on average −9.22%; individual values ranged from −3.3% to −16.9%). To further evaluate these vibratory effects (Figure 7), we analyzed the estimation error ([observer’s time estimation—agent’s walking time]/agent’s walking time x 100]). We found a significant effect for forward locomotion (F_(2.16)_ = 26.8, *p* < 0.0001, *η*^2^ = 0.77). Observers accurately estimated the agent’s walking speed in the No-vibration and TA-vibration conditions (*p* > 0.9), but not in the H-vibration condition (*p* < 0.001). We found comparable results for the backward locomotion (F_(2.16)_ = 28.1, *p* < 0.0001, *η*^2^ = 0.78).

**Figure 6:**
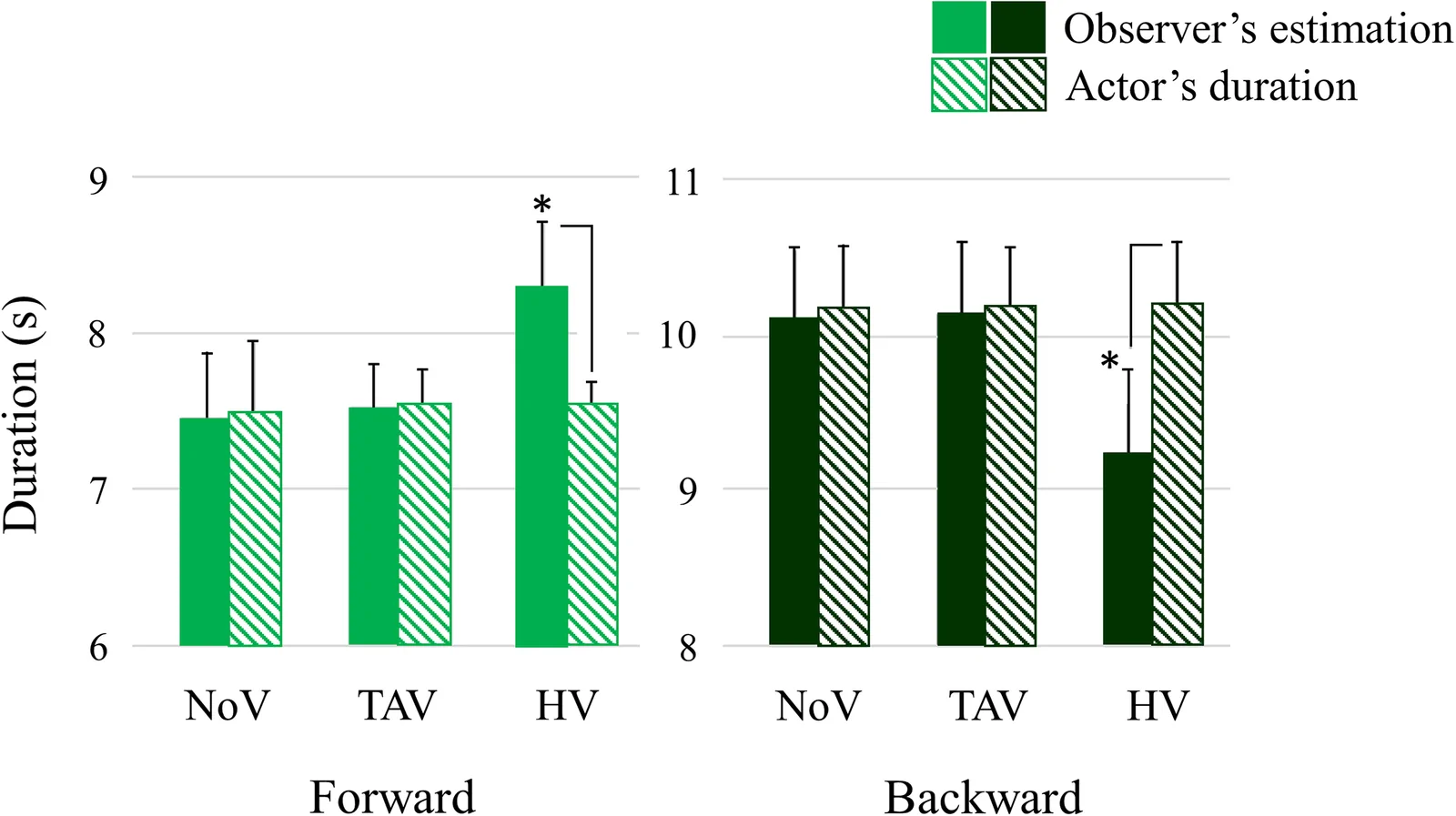
Experiment 3 Forward (light green) and backward (dark green) locomotion inference for each vibration condition. Results are expressed in terms of mean (+SD) durations (time in seconds estimated by the observer and performed by the actor on the 9 m linear path). Full bars represent the observer’s estimation, while striped bars show the real duration of the agent’s movement. Black lines connecting two bars indicate significant differences between the observer’s estimation and the actual actor locomotion duration. Stars indicate significant differences between the HV condition and the two other vibration conditions (NoV and TAV), within the direction of locomotion (forward and backward). NoV, no vibration; TAV, tibialis anterior vibration; H, hamstring vibration.

**Figure 7:**
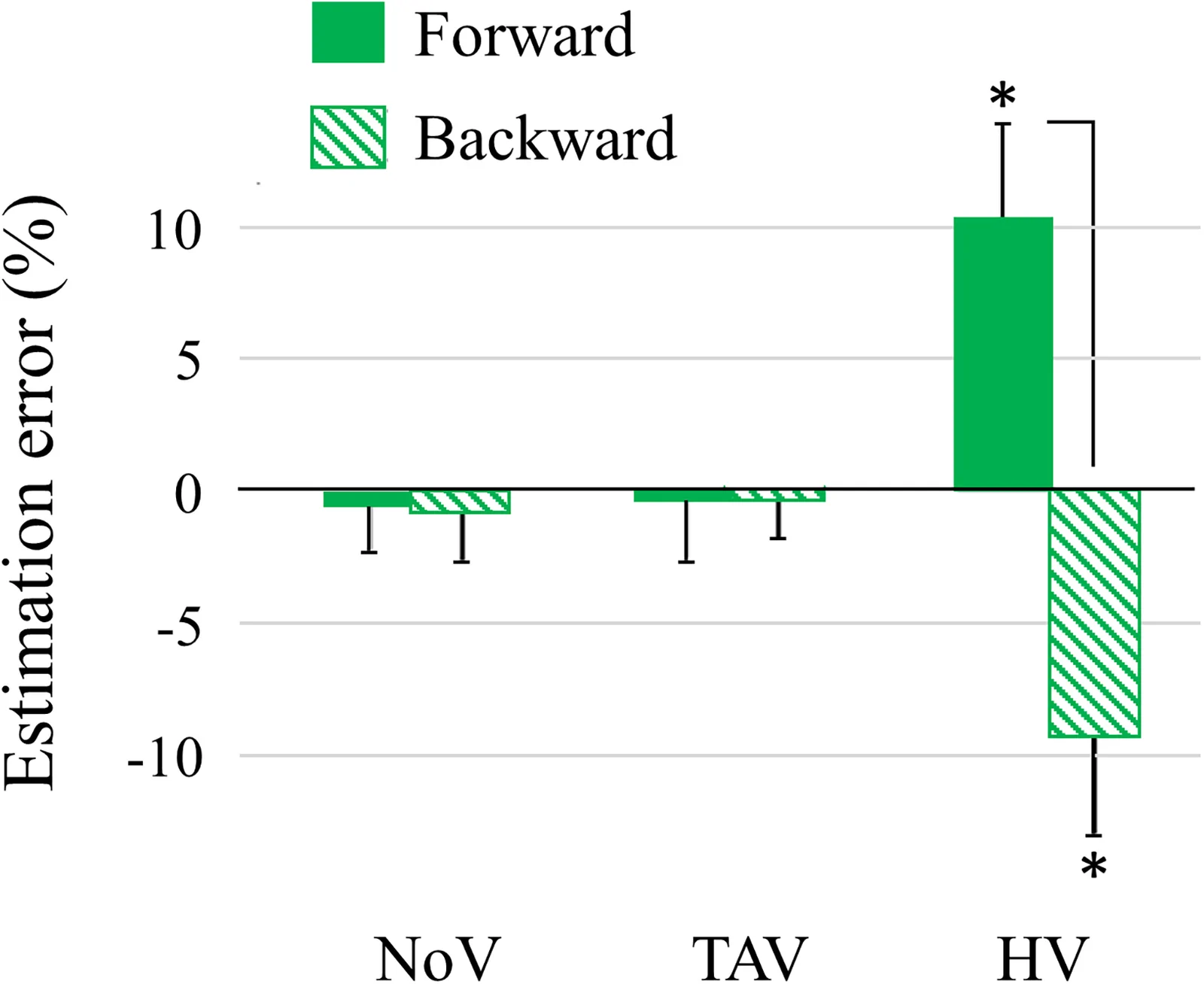
Experiment 3 Mean (+SD) estimation error (observer’s time estimation – actor walking time/observer’s time estimation x 100) for forward (green) and backward (striped green) locomotion inference, and each vibration condition. Black lines indicate significant differences between walking directions. Stars indicate significant differences between the HV condition and the two other vibration conditions (NoV and TAV), in forward and backward locomotion, respectively. NoV, no vibration; TAV, tibialis anterior vibration; H, hamstring vibration.

As for the other Experiments, there was no trial-by-trial modulation (for all conditions, t < 1 and *p* > 0.1, see Figure S1C). Note that hamstring muscle vibration at 40 Hz (control Experiment 3a, see Table S1) did not alter the temporal estimation of the agent’s speed during forward (permutation test; *t* = 0.04, *p* = 0.96, *d* = 0.01) and backward locomotion (permutation test; *t* = −0.16, *p* = 0.84, *d* = 0.05).

### Comparison of locomotion observation and imagined locomotion

Hamstring muscle vibration induced nearly opposite effects between the two tasks (Figure 8). In fact, the statistical comparison of the vibratory effects, assessed by the ratio [(HV – NoV)/NoV∗100)] and calculated separately for locomotion observation and mental locomotion, showed a significant impact for the forward (permutation: diff = −19.5, *p* < 0.01, *d* = −3.41) and backward locomotion (permutation: diff = 16.18, *p* < 0.01, *d* = 4.77).

**Figure 8:**
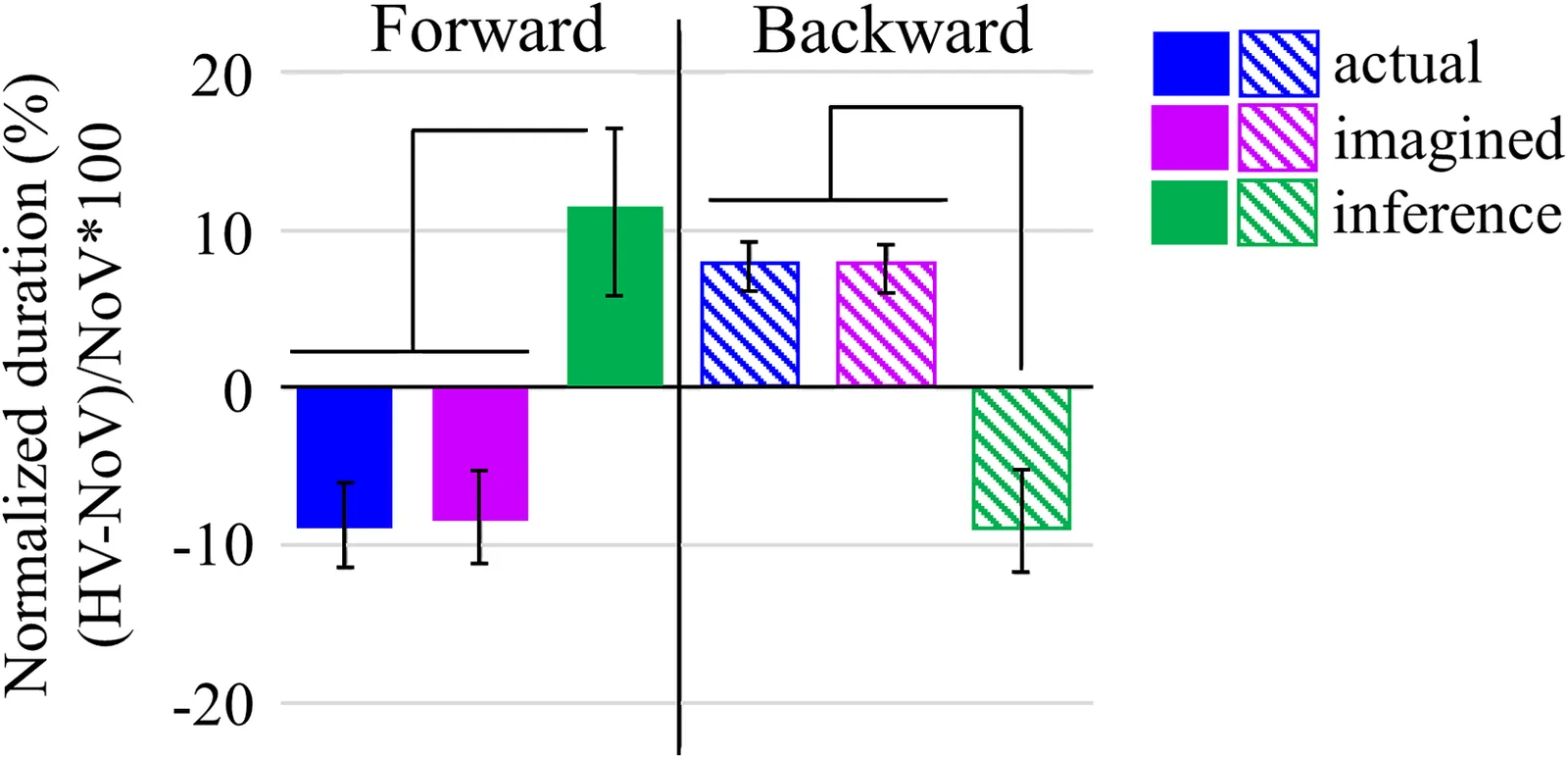
Effects of hamstring muscle vibration (normalized to no vibration, NoV), on actual locomotion, imagined locomotion, and locomotion inference, for forward and backward walking Black lines connecting two bars indicate significant differences between the conditions within each direction of locomotion. Note that histograms illustrating actual and imagined locomotion are the same as those of Figure 4A. This allows qualitative comparison with locomotion inference.

### Experiment 4: Locomotion observation under different conditions of visual feedback

In this Experiment, we hypothesized that the amount of available visual feedback would significantly influence motor prediction and, consequently, the observers’ ability to accurately estimate the duration of the agent’s locomotion. To test this idea, we recruited six new participants and conducted a similar experiment with some design modifications: the agent walked only forward, and only the hamstring muscle of the observers was vibrated.

In the first condition, similar to Experiment 3 (NoV), the agent walked at each observer’s natural speed, and the observer perceived the first third of the path without vibration. In the second condition (new), the agent increased his speed above the observer’s natural pace, while the observer again perceived only the first third of the path without vibration. The third condition (similar to Experiment 3, HV) involved the agent walking at the observer’s natural speed, but this time the observer perceived the first third of the path under hamstring muscle vibration (H-vibration). In the fourth condition (new), the agent walked at the “estimated” speed provided by the observer under H-vibration in the previous condition, with the observer still perceiving the first third of the path under vibration. Finally, in the fifth condition (new), the agent walked at the observer’s natural speed, but the observer perceived 2/3 of the path (6 m) while being vibrated. Each participant completed 12 trials for each condition, and the walking durations of both the agent and the observers were recorded as described in Experiment 3.

As instructed, the agent increased his walking speed in the “higher speed” and “matched speed” conditions (Figure 9). Permutation tests on the agent’s forward walking durations confirmed that the two conditions differed significantly from the three “natural speed” conditions (all *p* < 0.05). However, there was no significant difference between the “higher speed” and “matched speed” conditions (*p* = 0.52, *t* = −1.63, *d* = 0.54), confirming that the agent adhered to the task instructions.

**Figure 9:**
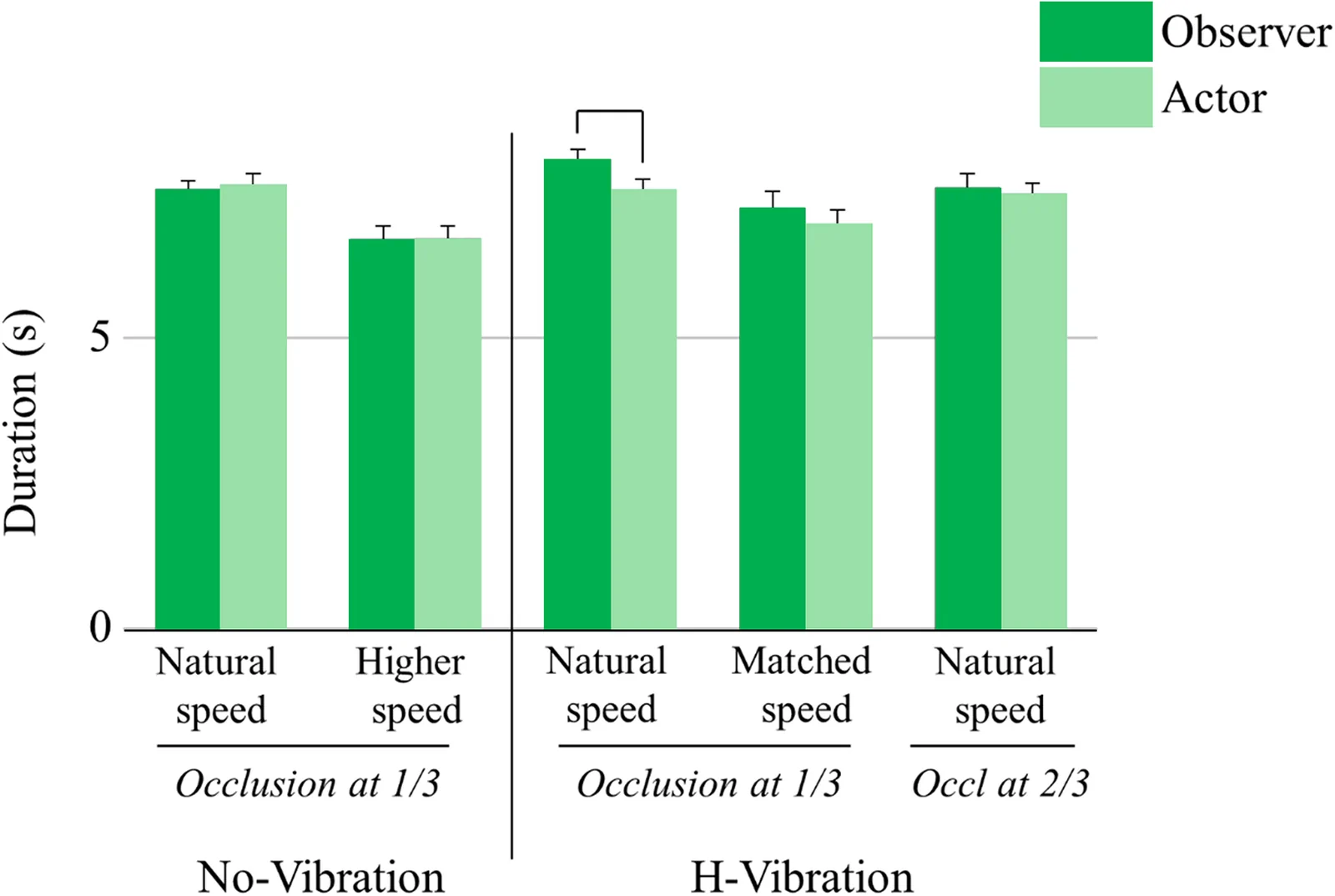
Experiment 4 Mean (+SD) duration of the actor’s locomotion (light green) and mean (+SD) durations estimated by the observer (dark green), across the five experimental conditions of the forward locomotion inference task. In conditions 1 and 2, no vibration (NoV) was applied to the observer, while in conditions 3 to 5, a hamstring muscle vibration (HV) was applied. In the “Natural speed” conditions, the actor walked at the observer’s natural speed. In the “Higher speed” condition, the actor walked at a speed 15% faster than the natural speed. In the “Matched speed” condition, the actor walked at the speed of the observer under HV. Finally, the timing of the visual occlusion for the observer was manipulated (occluded after 1/3 of the movement for conditions 1 to 4, and after 2/3 of the movement for condition 5). Black lines connecting two bars indicate significant differences between the real duration of the walk (actor’s walk duration) and the duration estimated by the observer.

For the observers, we replicated the effects seen in Experiment 3. Specifically, they accurately estimated the agent’s walking speed without vibration (Condition 1: ‘Natural speed, 1/3′ without vibration; *p* = 0.95, t = −0.52, *d* = 0.17), but overestimated it under vibration (Condition 3: ‘Natural speed, 1/3′ with H-vibration; *p* < 0.01; *t* = 5.31, *d* = 1.77). In Condition 2 (‘Higher speed, 1/3, without vibration’), we found that when the agent increased their walking speed, observers still estimated the speed accurately (*p* = 0.97, *t* = −0.47, *d* = 0.16), suggesting that locomotion observation was speed-independent in the absence of vibration. More intriguingly, in Condition 4 (‘Matched speed, 1/3, vibration’), when the agent’s speed matched the observer’s estimated speed under hamstring muscle vibration (i.e., the agent was walking faster), the observers’ estimates were accurate (no underestimation; *p* = 0.31, *t* = 1.90, *d* = 0.63). Compared to Condition 3 (‘Natural speed, 1/3, vibration’), where observers underestimated the agent’s speed, this novel finding indicates that the initial mismatch between the observer’s speed prediction and the agent’s actual speed creates an internal error signal that helps adjust speed estimation during locomotion inference. Finally, in Condition 5 (‘Natural speed, 2/3, vibration’), speed underestimation disappeared (*p* = 0.75, *t* = 0.99, *d* = 0.33) when observers could perceive the agent’s movement for a longer duration (6 m). In line with the *predictive* hypothesis, the increased visual feedback enabled the observer to refine their prediction, which closely aligned with the agent’s actual walking speed.

### Simulations

We employed a simplified state-space model (see STAR Methods for details) to simulate participants’ temporal estimations of the agent’s movement under different feedback conditions within the action observation protocol.

First, we ran a basic simulation to estimate the observer’s temporal prediction assuming complete feedback (i.e., continuous visualization of the agent’s entire movement). Based on an average duration of 7.5 s (derived from experimental results), we defined 18-time steps of 416 ms each. We set the initial prediction error at +10% of the actual duration and iteratively computed a new estimation at each subsequent time step. The error was adjusted by +10% to reflect the experimentally observed difference between the non-vibration and vibration conditions (Figure 9). The outcome of this simulation is shown in Figure 10 (red curve). Starting with an initial prediction error of 10%, the error decreased to below 5% after 9-time steps and to less than 1% after 15-time steps. This simulation closely mirrors the real data, as participants provided accurate speed estimations in Condition 5 of Experiment 4, where they observed the agent’s movement under vibration over 6 m (2/3 of the path).

**Figure 10:**
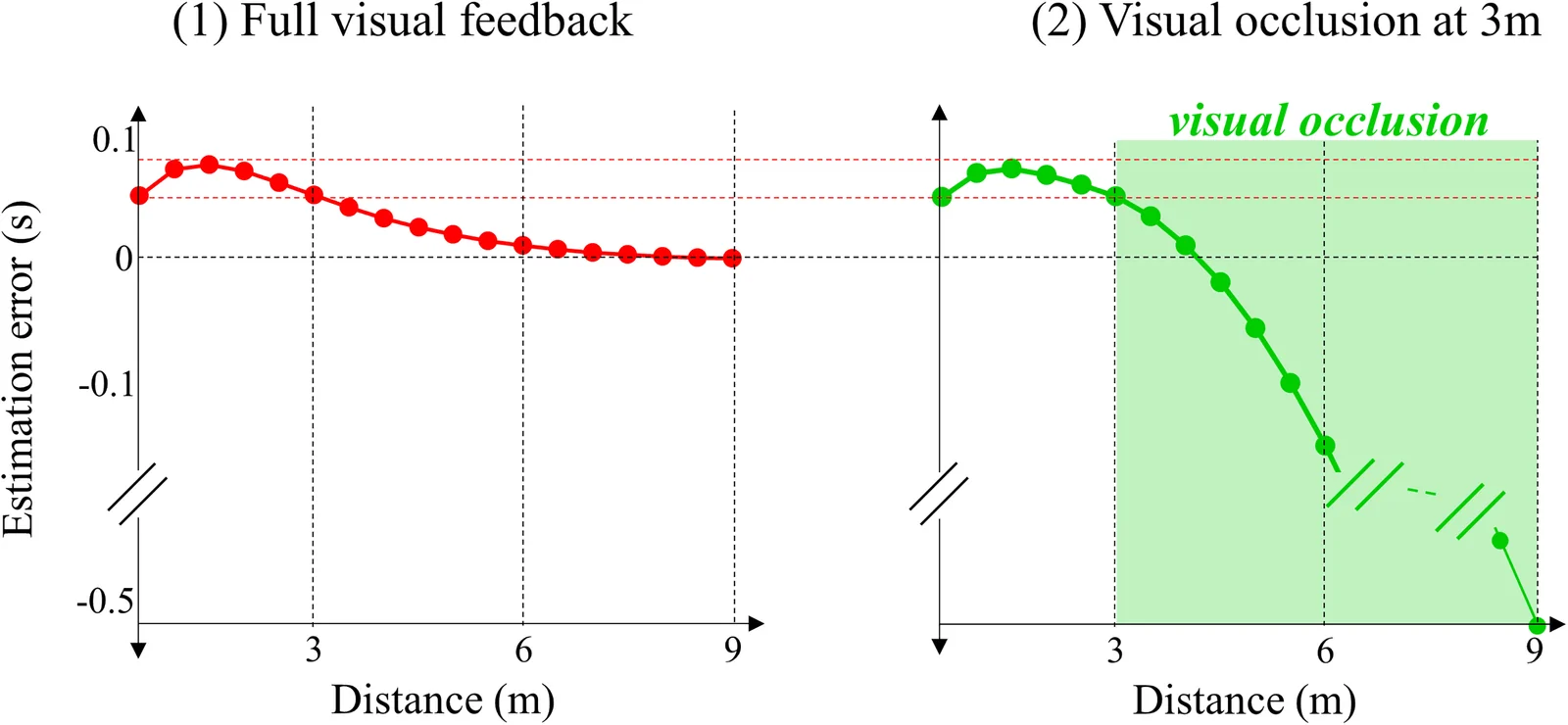
Results of the simulations for the forward walk The red and green curves represent the simulated duration estimated by the observer for the actor’s 9-meter walk under different visual contexts (without or with visual occlusion). These simulations used a simplified state space model (see STAR Methods for details). In (1), a basic simulation was run to estimate the observer’s temporal prediction with full visual feedback (continuous observation of the entire movement). Starting with an average duration of 7.5 s, we defined 18-time steps of 416 ms each. The initial prediction error was set at +10% of the actual duration and iteratively adjusted to reflect the experimentally observed difference between non-vibration and vibration conditions. In (2), we introduced visual occlusion at 3 m, preventing further updates to the prediction error. Once occluded, the prediction error remained constant, leading the observer to estimate the remaining 6 m as longer or shorter depending on the direction of the observed movement, as no new visual data were available to refine the estimate.

In the second simulation (Figure 10, green curve), we used the same +10% initial error but introduced visual occlusion at 3 m (i.e., restricted visual access to the agent’s movement, preventing further prediction error updates, as in Experiment 3). We assumed that once visual information was occluded, the prediction error would remain constant thereafter, as no new visual data would be available to refine the temporal prediction. If the observer continued to correct their prediction based on this last known error, the estimation error would eventually approach zero but would not stabilize. Instead, it would progress toward negative values, leading the observer to estimate the remaining 6 m as either longer (for forward locomotion) or shorter (for backward locomotion), depending on the direction of the observed movement.

Finally, we replicated these simulations for backward walking (Figure 11). Our results show that these simulations yield outcomes consistent with, but opposite in sign to, those obtained during forward motion (Figure 10). Notably, the blue curve (Figure 11) crosses the zero line and then continues to rise, reaching significant positive values.

**Figure 11:**
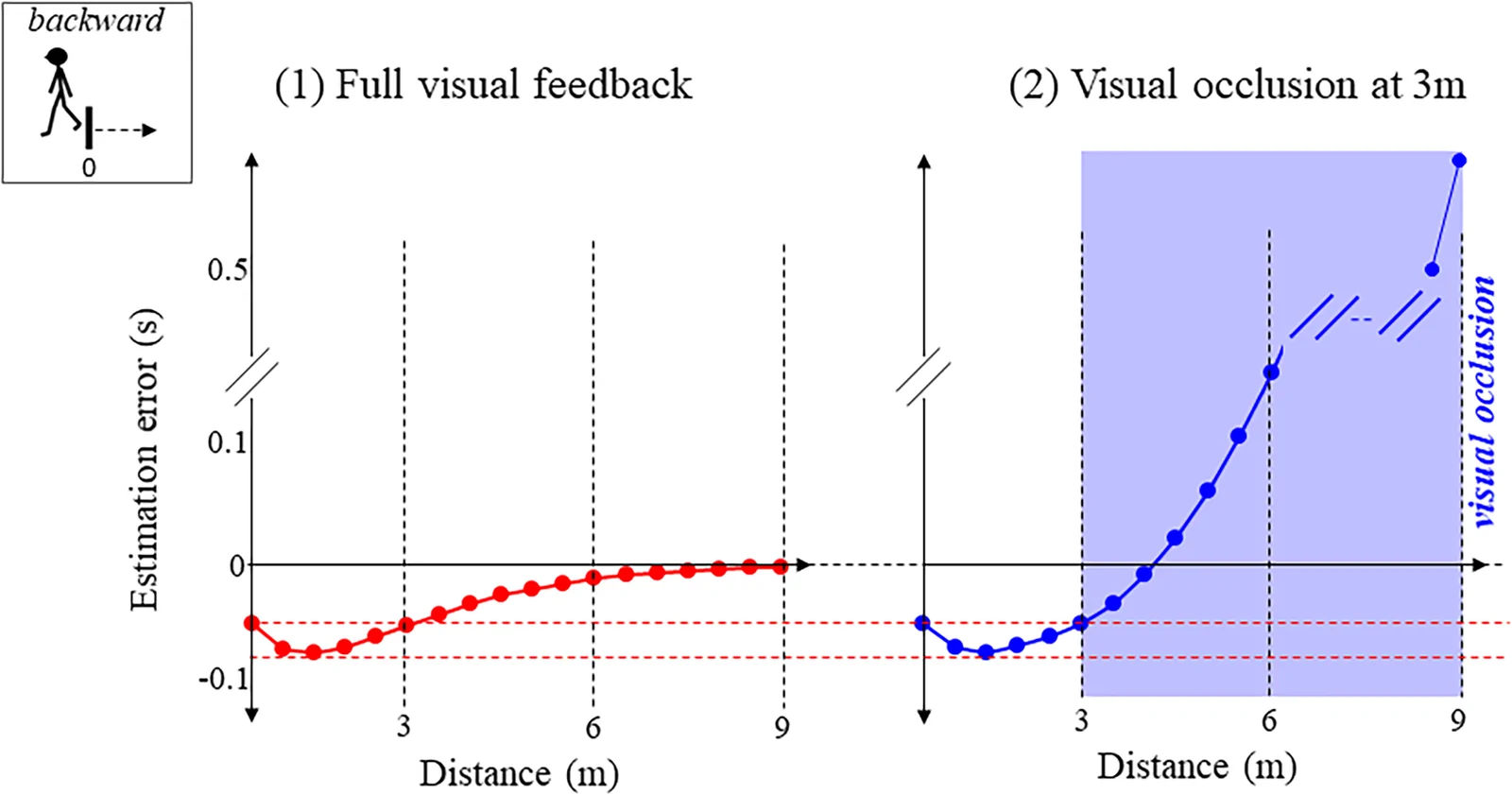
Results of the simulations for the backward walk The red and blue curves represent the simulated duration estimated by the observer for the actor’s 9-meter walk under different visual contexts (without or with visual occlusion). We replicated the previous simulations to accommodate the case of backward walk. We retained the same model and factors as before, with the only adjustments being the duration of the actor’s movement—8.5 s, divided into 18 steps of 47 ms each—and altering the initial error direction to −10% to reflect the actual error observed during backward walking.

These simulations provide a clearer understanding of our results. Without vibration, the observer’s perceived speed closely matches the agent’s actual speed. Hence, the first 3 m of observation would suffice to generate an accurate prediction (with an estimation error near zero). After visual occlusion, this correct prediction would reliably forecast the agent’s arrival time (similar to Condition 1 in Figure 10/Experiment 5). However, under vibration, the observer’s perceived speed deviates from the agent’s actual speed (similar to Condition 2 in Figure 10/Experiment 3, with an initial error of +10%). In this scenario, the first 3 m would not be sufficient to make an accurate prediction. As described, the observer would retain the last prediction error, unable to update it with new visual information, leading to either an overestimation or an underestimation of the agent’s speed (and, consequently, the duration of their movement).

## Discussion

In this study, we experimentally tested two competing hypotheses—*direct matching* and *predictive*—to understand the mechanisms underlying action observation. The *direct matching* hypothesis posits a direct correspondence between the visual perception of an agent’s movement and the activation of corresponding motor representations in the observer’s motor cortex, without any subsequent comparison process (see Figure 1A). In contrast, the *predictive* hypothesis suggests that, during action observation, sensorimotor predictions are generated and then compared with the visual information of the agent’s movement (see Figure 1B). To distinguish between these hypotheses, we designed a locomotor task under various experimental conditions. Participants were asked to either imagine walking—a well-established method for assessing motor prediction^33^^,^^34^—or to observe an agent walking and estimate the moment they would reach the end of the path (locomotion inference, as in^44^^,^^45^). We further manipulated both imagined and observed locomotion by inducing sensory illusions through peripheral mechanical muscle vibration. Our findings revealed that muscle vibration accelerated imagined forward locomotion and slowed imagined backward locomotion compared to non-vibrated conditions. Similar results were obtained for actual walking. The results were reversed during locomotion inference: observers estimated longer durations for an agent walking forward and shorter durations for an agent walking backward. To model these results, we employed a simplified state-space model.^46^^,^^47^ Collectively, our findings support the involvement of a *predictive* process in action observation.

A key aspect of motor behavior is the ability to predict the outcomes of actions before sensory feedback becomes available. This capability is thought to rely on internal forward models, which are neural networks that mimic the causal flow of physical processes by predicting future sensorimotor states (e.g., position, velocity) based on the efferent copy of the motor command and the current state.^23^^,^^36^ Such motor predictions are crucial for motor learning, particularly through mental practice.^48^^,^^49^^,^^50^^,^^51^ It has been proposed that the temporal characteristics of mental movements arise from these sensorimotor predictions generated by the forward model.^38^^,^^39^ In this study, we demonstrated that sensory illusions induced by muscle vibration produced specific directional effects on the timing of imagined locomotion compared to the non-vibration condition: forward locomotion was accelerated, while backward locomotion was decelerated. This is plausible because, during motor imagery, we internally simulate or predict upcoming sensory experiences (such as position and speed) based on motor commands that are prepared but not executed. Supporting this idea, research has shown that both motor imagery and the illusory sensation during arm movements activate common neural regions, including the contralateral cingulate motor areas, the supplementary motor area, the dorsal premotor cortex, and the ipsilateral cerebellum.^42^ In addition, previous studies have proposed that the combination of a proprioceptive evoked sensation with an imagined movement generates movements that are the sum of the vectors of the sensations evoked by the two modalities.^41^^,^^42^^,^^43^^,^^52^

Notably, similar results occur during real locomotion. This indicates that sensory illusions and the consistent effects of muscle vibration on movement are caused by predictive mechanisms operating in both actual and imagined actions, rather than by nonspecific factors such as an additional load that could influence attention or disrupt movement execution. Local muscle vibration stimulates sensory receptors and, in addition to creating sensory illusions, evokes short-term plasticity in the spinal cord and cortex.^53^^,^^54^ This raises the hypothesis that sensory afferences could affect the timing of both actual and imagined locomotion. However, such an effect would likely be global rather than specific to the direction of locomotion and might differ between actual locomotion (where motor commands are executed) and imagined locomotion (where motor commands are only prepared). The results of control experiments 1a and 1b support this conclusion.

We found that muscle vibration at 85 Hz produced a specific directional effect during action observation. Interestingly, this effect was reversed compared to imagined locomotion: participants overestimated the duration of forward walking and underestimated that of backward walking when observing an agent. This reversal illustrates a key aspect of prediction, where perception arises from comparing incoming sensory input with internal predictions. In Experiments 1 and 2, vibration biased the internal estimates of movement dynamics, leading to shorter durations during both imagined and actual walking.

In contrast, in Experiment 3, where participants observed someone else walking, the same biased prediction was compared to the agent’s actual movement. When the predicted movement was faster than the observed movement, a mismatch occurred, making the agent appear slower and thus lengthening the perceived duration. This dissociation shows that although vibration altered internal predictions similarly across tasks, the perceptual consequences differed: during imagery, predictions directly shaped temporal estimates; during observation, they served as a reference point, against which the agent’s movement was evaluated.

These results suggest that action observation engages active sensorimotor prediction mechanisms rather than merely passive resonance. The lack of an effect at 40 Hz, which does not induce proprioceptive illusions, confirms that the observed biases stem from altered sensorimotor predictions rather than from nonspecific effects of vibration. Moreover, the contrast between imagery and observation highlights the flexible role of internal models: in imagery, predictions operate in isolation; in observation, they are continually tested against visual input, allowing prediction errors to emerge. This dynamic use of internal predictions aligns with the prediction framework. Prior studies have shown that muscle vibration can modulate neural responses during action observation depending on the vividness of the illusory experience.^55^ Our findings extend this by showing that proprioceptive manipulations can systematically bias the temporal perception of others’ actions.

Two main theoretical concepts can be evoked to explain and understand how the human brain operates in action observation.

The *direct matching* hypothesis (Figure 1A) posits that mirror neurons map observed actions into motor representations, creating an automatic motor resonance in the frontoparietal areas.^14^ According to this hypothesis, a direct correspondence exists between the visual information extracted from an observed movement and the neural activations in the observer’s motor cortex that would facilitate producing the same movement. Essentially, there is a direct link between visual input and motor output.^16^^,^^18^ If this hypothesis applied to our study, we would expect different results from those we obtained. Specifically, sensory illusions would be unlikely to affect locomotor inference. If an automatic resonance mechanism were at play, it would directly capture the agent’s movement, meaning that the vibrations applied to the observing subject should not specifically alter the inference outcomes. At most, the vibration might produce a general effect, such as an additional attentional load, but not directly change the inference. Moreover, under the direct matching hypothesis, the proposed mechanism would be similar to that used in motor imagery, where no comparison between observed and predicted movement is possible. As a result, we would expect similar outcomes across both our inference and motor imagery tasks. However, this is not what we observed.

In contrast, in the *predictive* hypothesis,^25^^,^^26^^,^^27^^,^^28^^,^^29^^,^^56^ predictions about future actions are generated for comparison with incoming visual inputs, as the action is observed. When observing forward locomotion under muscle vibration, the observer is in a sensory predictive state that accelerates walking speed (see imagined locomotion). Therefore, when comparing the agent’s speed to their speed prediction, the observers estimate that the agent is moving more slowly and infer a longer overall duration than in the condition without vibration. The same reasoning can be applied to backward locomotion. This directional effect suggests that, to estimate agents’ motion, the observers rely on an internal error signal resulting from comparing the agents’ walking speed with their speed prediction. In line with this idea, we know from fMRI studies^8^^,^^57^^,^^58^^,^^59^ that observing an action triggers activity in the circuitry involved in motor prediction and comparison, including subcortical structures (i.e., cerebellum, dentate nucleus, thalamus).

When actions become predictable, this prediction can replace the actual visual inputs. In this regard, a previous study^30^ found a significant correlation between the level of corticospinal excitability (TMS over M1) and the degree of similarity between the observer’s motor style and the observed movement. Interestingly, the correlation they found was negative, meaning the greater the similarity, the smaller the associated MEPs. This increase in MEP amplitude may reflect the error-predictive signal from the cerebellum to the motor cortex. The involvement of a prediction process in action observation implies the ability to extract subtle visual kinematics from the observed action and to compare them with internal kinematic predictions. Recent neurophysiological findings plead in favor of such a process by showing the activation of the lateral cerebellum in action observation and the impairment of cerebellar patients with spinocerebellar ataxia to discriminate subtle differences in the kinematics of observed limb movements.^8^^,^^31^^,^^60^

Furthermore, our simulations (Figure 10) show that one potential mechanism explaining our results is the comparison between kinematic predictions based on the observer’s intrinsic skills and the visual inputs conveying information about the actor’s movement. In simulation 1 (Figure 10), the observer had continuous access to the agent’s movement, allowing them to predict the end of the walk accurately. This prediction was gradually refined by comparing the observer’s internal representation of the movement—shaped by context, priors, and individual motor signatures—with the incoming visual information. The prediction was adjusted in real time based on the prediction error calculated from these two inputs. In contrast, when the agent’s movement is occluded from view, the absence of new visual information prevents further updates to the initial prediction. In this scenario (Figure 10, simulation 2), the observer’s prediction relies solely on the error calculated before the occlusion. Without vibration, the observer’s perceived speed closely matches the agent’s actual speed, so the first 3 m of observation are sufficient to generate an accurate prediction with minimal estimation error. After visual occlusion, this accurate prediction would reliably forecast the agent’s arrival time (as seen in Condition 1 of Figure 10/Experiment 5). Conversely, under vibration, the observer’s perceived speed deviates from the agent’s actual speed (similar to Condition 2 of Figure 10/Experiment 3, with an initial error of +10%). In this case, the first 3 m of observation do not provide enough information to develop an accurate prediction. Consequently, the observer retains the last prediction error, which cannot be adjusted in the absence of new visual input, leading to either an overestimation or underestimation of the agent’s speed and the duration of their movement.

Reinforcing this idea, the results of Experiment 4 favor comparison-based operations, similar to those described in the *predictive* approach. Specifically, when we annulated the observer’s temporal estimation error, i.e., the mismatch between the observer’s speed prediction and the agent’s actual speed, by increasing the agent’s speed to match that of the observer’s under the vibration condition (see situation 4), the observer’s estimation was accurate. Additionally, when the visual feedback about the agent’s motion was increased (situation 5), allowing the observer to update better his/her predictions, estimations of the agent’s speed were also accurate. These results indicate that the observer assesses the agent’s speed not simply by mimicking the agent’s movement but by comparing the observed action to his prediction. Also, our findings were speed-independent, as altering the agent’s speed did not influence the observer’s ability to estimate the agent’s motion accurately.

Although our results support the *predictive* hypothesis, alternative explanations must also be considered. Attentional modulation or increased cognitive load may have influenced task performance; however, the specifically directional effects of vibration (e.g., opposite outcomes for forward vs. backward movement) argue against nonspecific attentional or cognitive accounts. Biomechanical factors may affect executed locomotion under vibration, but they are unlikely to account for effects observed during imagined or inferred actions, where no overt movement occurs.

In conclusion, this study improves our understanding of the mechanisms underlying action observation. Our data support the idea that an internal prediction process, including a comparison between the observer’s state prediction and the external visual feedback, is involved in action observation. This makes action observation a dynamic process where internal predictions are continuously updated until they align with external visual information.

### Limitations of the study

Several limitations should be acknowledged. First, sample sizes were relatively small, although consistent with previous studies using similar paradigms and partly mitigated by multiple control experiments and converging experimental conditions. Second, the study relies primarily on behavioral measures and model-based interpretations, preventing direct conclusions about the underlying neural mechanisms involved in predictive processes during action observation. Finally, locomotion was tested in controlled laboratory conditions using a linear path, which may limit the generalization of these findings to more complex or naturalistic action observation contexts.

## Resource availability

### Lead contact

Further information and requests for resources should be directed to and will be fulfilled by the lead contact, Pauline M. Hilt (pauline.hilt@ube.fr).

### Materials availability

This study did not generate new unique reagents.

### Data and code availability

• Data: All data generated in this study are included in the article and its supplemental information. Additional raw data supporting the findings of this study are available from the lead contact upon request.
• Code: This paper does not report original code.
• Additional information: Any additional information required to reanalyze the data reported in this paper is available from the lead contact upon request.

## Acknowledgments

T Pozzo was supported by ANR (VIMACO-ANR-21-CE10-0009). NG was supported by the program “Amorçage” from Région Bourgogne Franche Comté (PARK-CognAction, 2021Y-08279). L.F. was supported by PRIN 2020 and EU grant PRIMI.

## Author contributions

All authors designed the study. N.G. and L.D. conducted the experiments. N.G., P.H., and C.P. analyzed the data. P.H. and C.P. developed the modeling approach. N.G., P.H., and C.P. wrote the first draft of the manuscript. All authors contributed to data interpretation and revised the manuscript critically. All co-authors have read and approved the final version of the manuscript.

## Declaration of interests

The authors declare that they have no competing interests.

## STAR★Methods

### Key resources table

**Table:** 

REAGENT or RESOURCE	SOURCE	IDENTIFIER
Human participants	Recruited at Université de Bourgogne	See Table S2
Mechanical vibrators (VB115)	Technoconcept, France	VB115
Crystal occlusion goggles (Plato system)	Translucent Technologies	Plato Visual Occlusion System
Surface EMG system (SMART)	BTS Bioengineering, Italy	SMART System
Silver-chloride bipolar surface electrodes	BTS Bioengineering, Italy	Standard surface EMG electrodes
Electronic stopwatch (1 ms resolution)	Commercial device	Not applicable
Clinical goniometer (Protractor Goniometer)	Prestige Medical	Standard clinical goniometer
MATLAB R2024a	MathWorks	https://www.mathworks.com

### Experimental model and study participant details

All participants gave written informed consent to join the study, which was approved by the Ethics Committee of the *Université de Bourgogne* (approval number: CERUBFC-2024-01-30-003) and was conducted following the Declaration of Helsinki. Participants were free from neuromuscular and cognitive disorders and were sensitive to vibratory stimulation from the first application. None of them had previous experience of the sensorimotor effects caused by musculotendinous vibration and were unaware of the specific hypotheses being tested in the study.

The number of participants was determined based on previous studies from our lab using similar experimental tasks.^33^ Therefore, based on published data showing locomotion durations of 6.05 ± 1.02 s for natural velocity and 4.03 ± 0.2 s for fast velocity, only 5 participants would be necessary to detect significant differences in movement durations, with a level of α = 0.05 and a power of 0.9 (Biostatgv software).

Age and sex distributions are provided in each experiment description and detailed in Table S2 Race and ethnicity data were not collected. Because of limited sample size, analyses were not powered to examine sex-related effects; this should be considered when generalizing the findings. This study does not meet the NIH definition of a clinical trial.

### Method details

We used a series of behavioral experiments to test how vibration-induced sensory illusions influence motor prediction and action observation. Experiments 1 and 2 examined imagined and executed locomotion, respectively, whereas Experiment 3 investigated temporal inference during locomotion observation. Experiment 4 manipulated visual feedback to further probe prediction-based mechanisms. Control experiments were included to rule out nonspecific effects of vibration.

All analyses were performed in MATLAB R2024a. Data are reported as mean ± SEM unless otherwise stated. The sample size (n) refers to the number of participants included in each experiment.

#### Experiment 1: Imagined locomotion

Nine volunteers (5 females, 4 males, mean age: 25.4 ± 2.7 years) participated in this Experiment, which took place in a quiet and large room (10 x 20 m), temperature regulated (22 ± 2°C), and illuminated with homogeneous white light. The floor was smooth and plain. On the floor, two black lines marked the start and the end point of a walking path (9 meters). Participants stood upright behind the starting line, their arms hanging by their sides, and their feet parallel and slightly apart. They were requested to mentally simulate (motor imagery) walking at a natural speed along the path in either a forward or backward direction, with eyes open to facilitate the visualization of the path, and under three vibration conditions: No-Vibration (NoV), bilateral vibration of the tibialis anterior muscles (TAV), and bilateral vibration of the hamstring muscles (HV).

We instructed participants to imagine performing the motor task from a first-person perspective (i.e., motor or kinesthetic imagery, as in^61^^,^^62^), rather than just visualizing themselves doing it (visual imagery). The participants completed 12 trials in each experimental condition (*n* = 72; 12 trials x 2 walking directions x 3 vibrations). The vibration conditions (NoV, TAV, and HV) were spaced out by at least twenty-four hours, to avoid interferences due to the post-effects of muscle vibration^63^^,^^64^ and were counterbalanced among the participants.

Within each vibration condition, forward and backward locomotion was also counterbalanced among the participants. The duration of mental movements was recorded using an electronic stopwatch (temporal resolution 1 ms) that participants held in their left arm. They started the stopwatch when they mentally began walking and stopped it when they mentally reached the end line. This mental chronometry paradigm is known to provide reliable results (for example^65^^,^^66^^,^^67^). The experimenter only had access to the recorded durations so participants had no information about their imagined walking speed. Mechanical vibration began 5 s before the onset of the imagined movement and ended 5s after its achievement. All the participants verbally reported at the end of the Experiment that they imagined walking in a first-person perspective without difficulties. Additionally, after each block of imagined trials, the participants reported the subjective estimation (SE) of the imagined movement quality through a 7-point Likert scale (1: very hard to feel and see the movement, 7: very easy to feel and see the movement, 2–6: intermediate score). Imagery ability was also assessed during brief pre-experimental sessions, where we confirmed similar durations between imagined and executed movement for simple motor tasks (i.e. writing, pointing).

#### Control experiment 1a

To examine the effects of vibration frequency on mental locomotion, Experiment 1 was repeated with the muscles’ vibration set at 40 Hz this time. Five participants who had previously taken part in Experiment 1 (2 females, 3 males, mean age: 24.4 ± 2.1 years) participated in this control. This control was always carried out two days after Experiment 1.

#### Control experiment 1b

Muscle vibration of the tibialis anterior and hamstring muscles caused postural reactions (Figure 2). To control whether these postures could affect mental walking durations, we asked five participants, who had already taken part in Experiment 1 (1 female, 4 males, mean age: 23.9 ±1.8 years), to imagine forward walking without any muscle vibration, but by adopting the postures elicited by the vibration of the tibialis anterior and hamstring muscles. This control was always realized three days after Experiment 1.

#### Experiment 2: Actual locomotion

The nine participants of Experiment 1 took also part in Experiment 2, at least one week after their involvement in Experiment 1 and the control Experiments 1a and 1b. The experimental design was identical to that of Experiment 1 (i.e., 2 walking directions x 3 vibrations) and was realized in the same environment. Participants were requested to walk along the 9 m linear path, at a natural speed, with full vision. Walking along the path at self-paced velocity required at least five gait cycles. Participants accomplished 12 trials for each experimental condition. The walking duration was recorded using an electronic chronometer. Participants started the stopwatch when they started to walk and stopped it when they reached the end line. As in Experiment 1, the experimenter only had access to the recorded durations so participants had no information about their imagined walking speed. The mechanical vibration began 5 s before the onset of the locomotion and ended 5s after its achievement.

#### Control experiment 2

To control the effects of vibration frequency on actual locomotion, Experiment 2 was replicated by setting the muscle vibration at 40 Hz this time. Five participants from Experiment 2 (2 females, 3 males, mean age: 23.1± 3.0 years) took part in this control.

#### Experiment 3: Locomotion observation

A new group of nine volunteers (4 females, 5 males, mean age: 25.2 ± 2.1 years), novices in the effects of muscle vibration on actual and mental locomotion, participated in Experiment 3, which took place in the same environment as the two others. Each participant (hereafter ‘observer’) was asked to estimate the duration needed by another human (hereafter ‘agent’) to walk along the same 9 m linear path. The agent walked forward or backward, with full vision, at a natural pace, without muscle vibration. Preliminary practice allowed the agent to adapt its speed to the natural speed of each observer with small variability. This was possible because, before the start of the Experiment, we asked the observers to naturally walk along the path (ten trials) while measuring the duration of their locomotion. The observers stood upright perpendicular to the side of the agent, at a distance of 2 m. They could fully perceive the agent's locomotion for one-third of the path (3 m). For the remaining part of the path (6 m), the observers’ view was occluded by crystal goggles with a wireless obstruction system (*Plato System*, 2 ms resolution). The agent’s walking duration and the corresponding temporal estimate by the observer were recorded using two electronic chronometers manipulated by the agent and the observer and synchronized via a computer program. Precisely, when the agent started to walk, he triggered both chronometers (his own and the observer’s). The agent stopped his chronometer at the end of the walk, and the observer stopped his/her chronometer when she/he estimated the end of the agent’s walk. The observer realized the task under the same vibration conditions (NoV, TAV, and HV) as in the previous Experiments. Vibrations were spaced out by at least twenty-four hours, to avoid interferences due to the post-effects of muscle vibration) and were counterbalanced among the participants. Within each vibration condition, forward and backward locomotion was also counterbalanced among the participants. The experimenter only had access to the recorded durations, so participants had no information about their temporal estimation. Mechanical vibration began 5 seconds before the onset of the agents’ locomotion and ended 5s after its achievement.

#### Control experiment 3a

To control the effects of vibration frequency on locomotion inference, Experiment 3 was repeated with the muscle vibration set at 40 Hz. Five new participants (1 female, 4 males, mean age: 21.8± 2.9 years) took part in this control.

#### Experiment 4: Motor inference of locomotion under different conditions of visual feedback

To further explore the mechanisms underlying locomotion inference, we recruited six new participants (2 females and 4 males, mean age: 23.9 ± 2.2 years) to perform the same Experiment with some modifications in the experimental design. Of the five conditions, the 1^st^ and 3^rd^ were similar between Experiments 3 and 4, while the other three were new.

In Experiment 4, the agent walked only forward, and only the observers’ hamstring muscles were vibrated. All conditions were conducted on different days and were counterbalanced among participants. Hamstring muscle vibration resembled previous experiments, and only the experimenter had access to the recorded durations. In the first condition (NoV, Natural speed, 1/3 of the path), the agent moved at the natural walking speed of each observer, who perceived the agent’s locomotion over 1/3 of the path without vibration. In the second condition (NoV, Higher speed, 1/3), the agent adopted a higher speed (+15%) than the natural walking speed of each observer, who perceived the agent’s locomotion over 1/3 of the path without vibration.

In the third condition (HV, Natural speed, 1/3 of the path), the agent adopted each observer’s natural walking speed, with the observer perceiving the agent’s locomotion for 1/3 of the path while being vibrated. In the fourth condition (HV, Matched speed, 1/3), the agent adopted the ‘*estimated’* walking speed of each observer; that is, the agent walked at the speed that the observer indicated while being vibrated (see previous condition). The observer perceived the agent’s locomotion for 1/3 of the path while being vibrated. In the fifth condition (HV, Natural speed, 2/3), the agent adopted the natural walking speed of each observer, who perceived the agent’s locomotion for 2/3 of the path while being vibrated. Each participant realized 12 trials in each condition.

The durations of the agent and observers were recorded as described in Experiment 3. The desired walking durations of the agent were determined through preliminary practice trials, allowing for precise adjustment of its walking pace. Indeed, for each condition, the agent was given a specific target duration before performing the walking task; then, its actual walking duration was compared to the target duration. The agent could then recursively adapt its speed in the following trial until it reached the right duration (± 0.2 s). This trial-and-error strategy allowed us to obtain the desired durations after 3 to 4 attempts. The agent’s walking durations across all vibration conditions were highly reliable and reproducible (see supplemental information, Figure 5).

#### Movement duration recording

In all Experiments, the duration of mental, actual, and inferred locomotion was recorded using an electronic stopwatch (temporal resolution of 1 ms) that participants held in their left arm. The specific procedure is explained in each Experiment.

#### Muscle vibration

Tibialis Anterior and Hamstring muscles were bilaterally stimulated by two mechanical vibrators (VB115, Technoconcept, France, biaxial DC motors, 7 cm long, with a radius of 4.5 cm and a weight of 150 g, delivering a 1.5 mm amplitude/80 Hz sinusoidal signal). The vibrators were cautiously fastened to the participants’ limbs with elastic bands in a way that did not disturb natural locomotion or posture. The tension developed by the elastic bands (3 cm wide) was about 7 N cm^-1^, which corresponded to a pressure of about 1 N cm^-2^ (i.e., 75 mmHg acting on the arterial vessel walls, which is about 50% of the compression value necessary to occlude them while in standing position). The vibrators were fixed on the Tibialis Anterior (∼4 cm above the ankle joint) and the Hamstring distal (∼3 cm above the knee joint) tendons. These two muscles were selected after a pre-test session ran on five participants, who did not take part in the main Experiments. We tested the effects of vibration of Triceps Surae, Tibialis Anterior, Hamstring, and Splenius muscles on body posture and walking speed. Following previous studies,^40^^,^^68^^,^^69^ we found that vibration of the Triceps Surae and Tibialis Anterior muscles had a strong postural effect, namely a backward and a forward body tilt, respectively, but no effect on walking speed. On the other hand, the vibration of the Hamstring and Splenius muscles had both postural and walking effects. Hamstring muscle vibration elicited backward inclination of the trunk and forward inclination of the shank due to hip extension and knee flexion. Splenius muscle vibration induced a forward body tilt. In addition, both vibratory stimulations accelerated forward locomotion and decelerated backward locomotion. According to these preliminary results, and to alleviate the whole protocol, we decided to vibrate the Tibialis Anterior and Hamstring muscles during the main and control Experiments.

#### Postural evaluation

The effect of muscle vibration on body posture was assessed in each Experiment before completing the different locomotor tasks. We used a standard clinical goniometer (Protractor Goniometer, *Prestige Medical*) to measure specific joint angles, i.e., ankle and hip. Participants stood on a flat surface, with feet positioned shoulder-width apart and arms relaxed along the body. For the ankle, the angle between the lateral malleolus, fibular head, and fifth metatarsal was measured to quantify dorsiflexion/plantarflexion alignment. Trunk inclination was determined by measuring the angle between the acromion, greater trochanter, and a vertical reference line. All measurements were taken on the right side, three times each, and averaged for each vibration condition. A single trained examiner performed all assessments to reduce variability.

#### Electromyographic activity

We recorded the EMG activity (SMART system, BTS, Italy) of the lower limb muscles during Experiments 1 (Mental locomotion) and 3 (Locomotion inference), as well as during 12 rest trials where participants were erect and immobile for 8 s without performing any task. The EMG activity was recorded using two silver-chloride bipolar surface electrodes from eight muscle bellies: soleus, gastrocnemius medialis, gastrocnemius lateralis, tibialis anterior, biceps femoris, rectus femoris, vastus medialis, and vastus lateralis. A ground electrode was placed on the forehead. EMG signal was pre-amplified, digitized, and transmitted to the remote amplifier via an optic fiber. It was sampled at 1000 Hz, filtered between 20-400 Hz (Butterworth), and rectified for further analysis. To quantify muscle activation, we calculated the root mean square (RMS) values of the EMG signal, using the following formula:

(Equation 1) $$RMS=\sqrt{\frac{1}{T}\int_{t}^{0}{\left(EMG\right)}^{2}dt}$$

RMS values during all NoV trials were not significantly different from those of the rest trials (two-tailed paired t-tests with Bonferroni correction: soleus: t(11) = 0.83, *p* = 0.42; gastrocnemius medialis: t(11) = 1.12, *p* = 0.29; gastrocnemius lateralis: t(11) = 0.95, *p* = 0.36; tibialis anterior: t(11) = 0.77, *p* = 0.46; biceps femoris: t(11) = 1.05, *p* = 0.32; rectus femoris: t(11) = 0.91, *p* = 0.38; vastus medialis: t(11) = 0.88, *p* = 0.39; vastus lateralis: t(11) = 1.09, *p* = 0.30).

### Quantification and statistical analysis

All statistical analyses were conducted using MATLAB R2024a. The normality of the distribution of paired differences was assessed using the Lilliefors test. When the normality assumption was met (*p* > 0.05), we used parametric paired-sample t-tests. When normality was violated (*p* < 0.05), we applied non-parametric permutation tests on the mean of paired differences. For each permutation test, a surrogate distribution was generated by randomly flipping the signs of the paired differences across 10,000 iterations, and two-tailed *p*-values were computed as the proportion of permuted means greater than or equal to the absolute value of the observed mean. For all paired comparisons, we also calculated Cohen’s *d* as a measure of effect size.

For comparisons involving more than two related conditions, repeated-measures ANOVAs were used, with partial eta squared (η^2^) reported as the effect size. When necessary, Scheffé’s test was used for post hoc comparisons, as it offers robust control over Type I error across multiple contrasts. For all multiple comparison contexts, *p*-values were adjusted using Bonferroni correction unless stated otherwise. The significance threshold was set at α = 0.05 (corrected where applicable). Since no trial-by-trial modulation was detected in any condition, analyses were conducted on averaged values per participant and condition.

In Experiments 1, 2, and 3, we tested the effects of vibration condition (NoV, TAV, HV) and direction (forward vs. backward) using two-way repeated-measures ANOVAs, followed by Scheffé-corrected comparisons when appropriate. For Experiment 3, we also computed the estimation error ([(observer’s estimated time − agent’s actual time) / agent’s time] × 100) to assess the perceptual bias introduced by vibration directly. This dependent variable was submitted to two-way ANOVAs to test for the vibration × direction interaction and to explore direction-specific effects.

To compare the magnitude of vibration effects across tasks (e.g., imagined vs. actual or observed locomotion), we computed normalized change scores using the formula:

$$Vibration\;Effect=\frac{V-NoV}{NoV}\times 100$$

These scores were submitted to repeated-measures ANOVAs and used in Pearson correlation analyses (with Bonferroni correction) to assess the relationship between motor imagery and actual locomotion effects.

To evaluate potential adaptation or fatigue effects over time, we compared the average durations from early (trials 1–3) and late (trials 10–12) trials in Experiments 1–3 using two-tailed paired t-tests, with a Bonferroni-adjusted significance level (α = 0.017).

Finally, for Experiment 4 and all control Experiments, where data were not normally distributed or sample sizes were small, we used permutation tests exclusively. All *p*-values were corrected for multiple testing using Bonferroni adjustment, and effect sizes were systematically reported.

#### Simulations

To confirm our hypotheses, we modeled the results that participants would theoretically achieve under different feedback conditions within the action observation protocol. To do so, we used a simple version of the state space model.^46^^,^^47^ In this version^70^ the motor adaptation that a participant makes to new incoming information can be described by the following equation:

(Equation 2) x(t)=Ax(t−1)−Be(t−1)

where x(t) represents the extent of adaptation at time sample t (i.e., the estimation of the agent’s movement duration at each step). Factor A defined the level of retention from one time step to the other. As our time samples are consecutive and last only a few milliseconds, we set the A factor to 1 to focus on the case of perfect retention. The term e(t-1) represents the error of estimation on the previous time step (t-1). The associated parameter B represents the learning rate (i.e. the proportion of error that will be compensated at the next time step). Here, we set B = 0.3.^71^

We ran a first simulation to obtain an initial estimate of the observer's prediction in the case where they would have complete feedback (visualisation of the total duration of the agent's movement). Based on an average duration of 7.5 seconds (duration deduced from experimental results), we defined 18-time steps of 416 milliseconds each. We estimated the initial error (first-time step) at 10% of the real duration and computed then iteratively the value of x at each time step, using the parameters defined above and the formula Equation 1. The error has been set at 10% to reflect the experimentally observed error between the condition without vibration and the condition with vibration (Figure 6).

In the second simulation (Figure 10, green curve), we started from the same 10% error. Still, we added a visual occlusion at 3m (i.e. no more access to visual information on the agent's movement and therefore no possibility of updating the prediction error). In this model, we consider this condition to be equivalent to keeping the prediction error constant (since no new visual information will update the prediction). Note that this simulation does not faithfully mimic our action observation protocol. In our protocol, the agent walked at the same speed as the subject. Since the initial prediction is supposed to be based on the subject's skills, its initial estimate should be very close to the expected value (error close to 0). The initial 10% error as defined in the second simulation could therefore represent a condition in which the subject's speed and the agent's speed are different, as is the case in our vibration condition.

Finally, we replicated these simulations to accommodate the case of backward walk. We retained the same model and factors as before, with the only adjustments being the duration of the actor's movement—8.5 seconds, divided into 18 steps of 47 milliseconds each—and altering the initial error direction to -10% to reflect the actual error observed during backward walking. The results of these simulations are shown in Figure 11.
