# Supplementary material for: Evidence for a sensorimotor prediction in action observation
Source: iScience. 2026 Apr 24;29(10):115876. doi: 10.1016/j.isci.2026.115876 (PMC13632192; doi:10.1016/j.isci.2026.115876)
Supplement: Supplementary file 1 [file mmc1.pdf]

iScience, Volume *29*

Supplemental information

Evidence for a sensorimotor prediction in action observation

Pauline M. Hilt, Nicolas Gueugneau, Laurent Demougeot, Luciano Fadiga, Thierry Pozzo, and Charalambos Papaxanthis

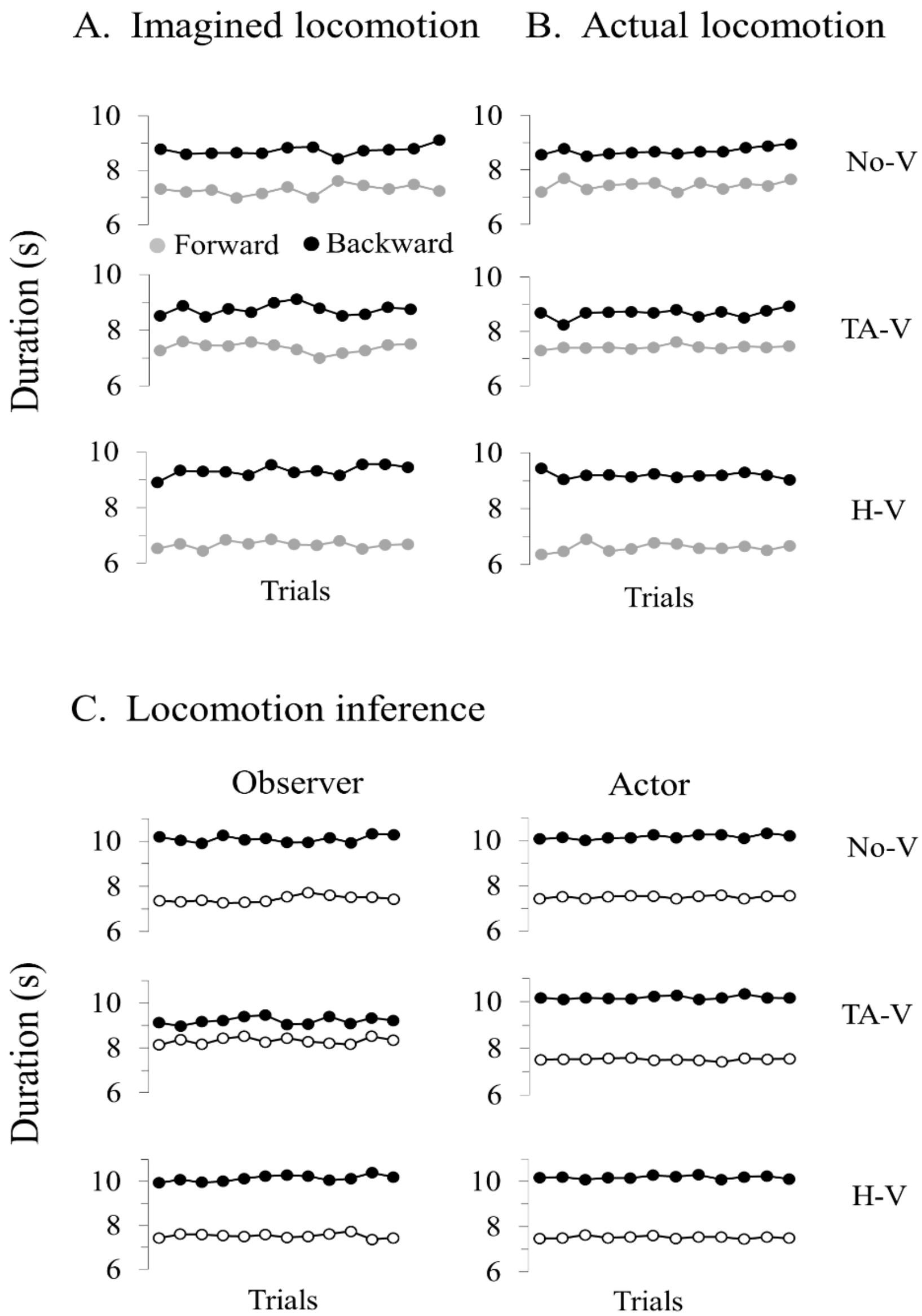

Figure S1: Trial-by-trial locomotion durations across vibration conditions, related to Results – Experiments 1-3. Mean trial-by-trial durations (s) for forward (gray) and backward (black) imagined locomotion (A, Experiment 1) and actual locomotion (B, Experiment 2) across vibration conditions. Panel C shows mean trial-by-trial observer estimations and actor movement durations during forward (white) and backward (black) walking in Experiment 3. Stability across trials was assessed by comparing the first three trials (1–3) with the last three trials (10–12), revealing no significant differences (all $t < 1$, $p > 0.1$). NoV, no vibration; TAV, tibialis anterior vibration; HV, hamstring vibration.

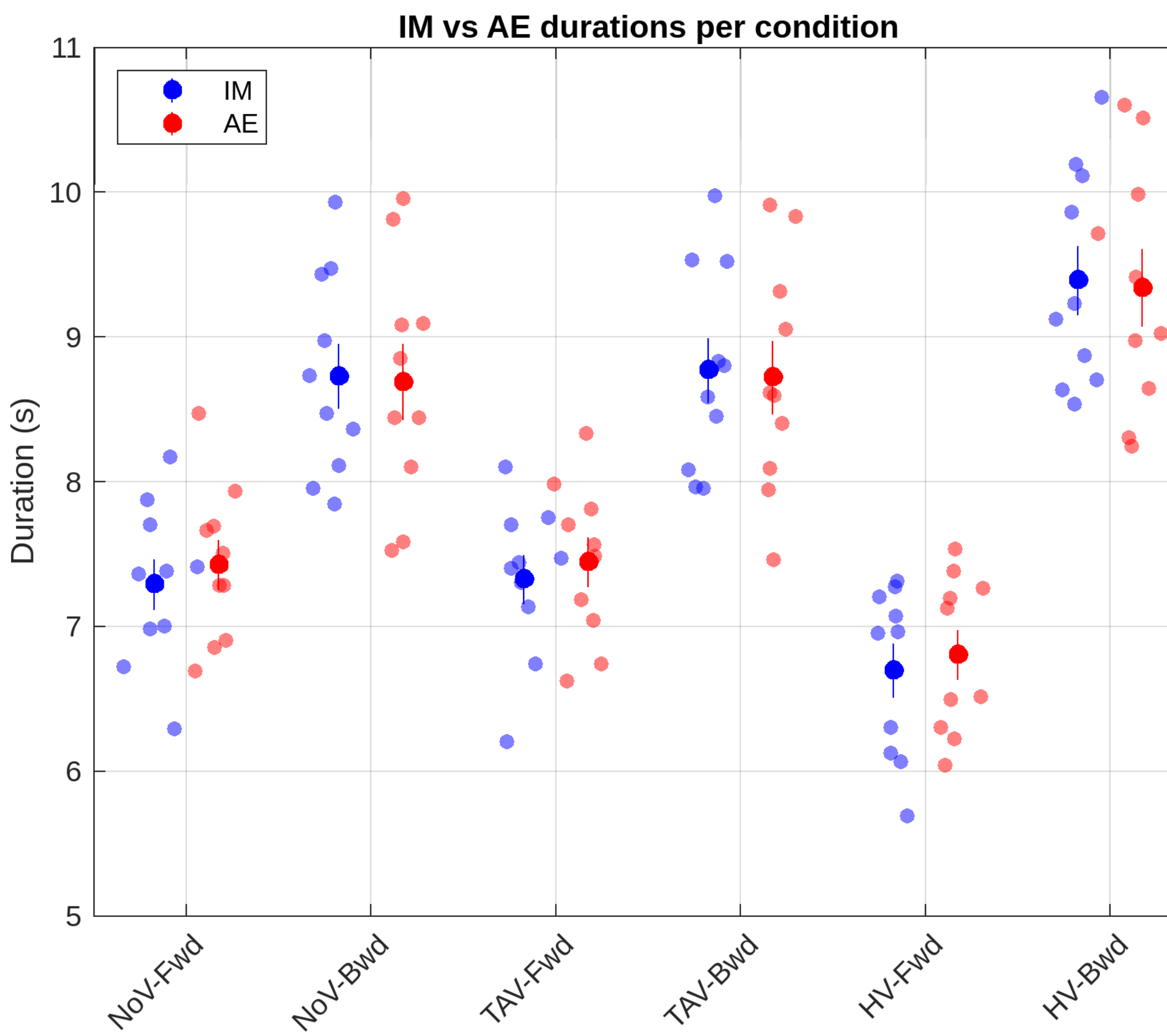

Figure S2: Imagined and executed locomotion durations across vibration conditions, related to Experiments 1–2. Mean locomotion durations (± SEM) during imagined locomotion (IM, blue) and actual execution (AE, red) across six experimental conditions combining vibration (NoV, TAV, HV) and walking direction (forward, backward). Dots represent individual participant values, illustrating the consistent effects of vibration across participants and tasks.

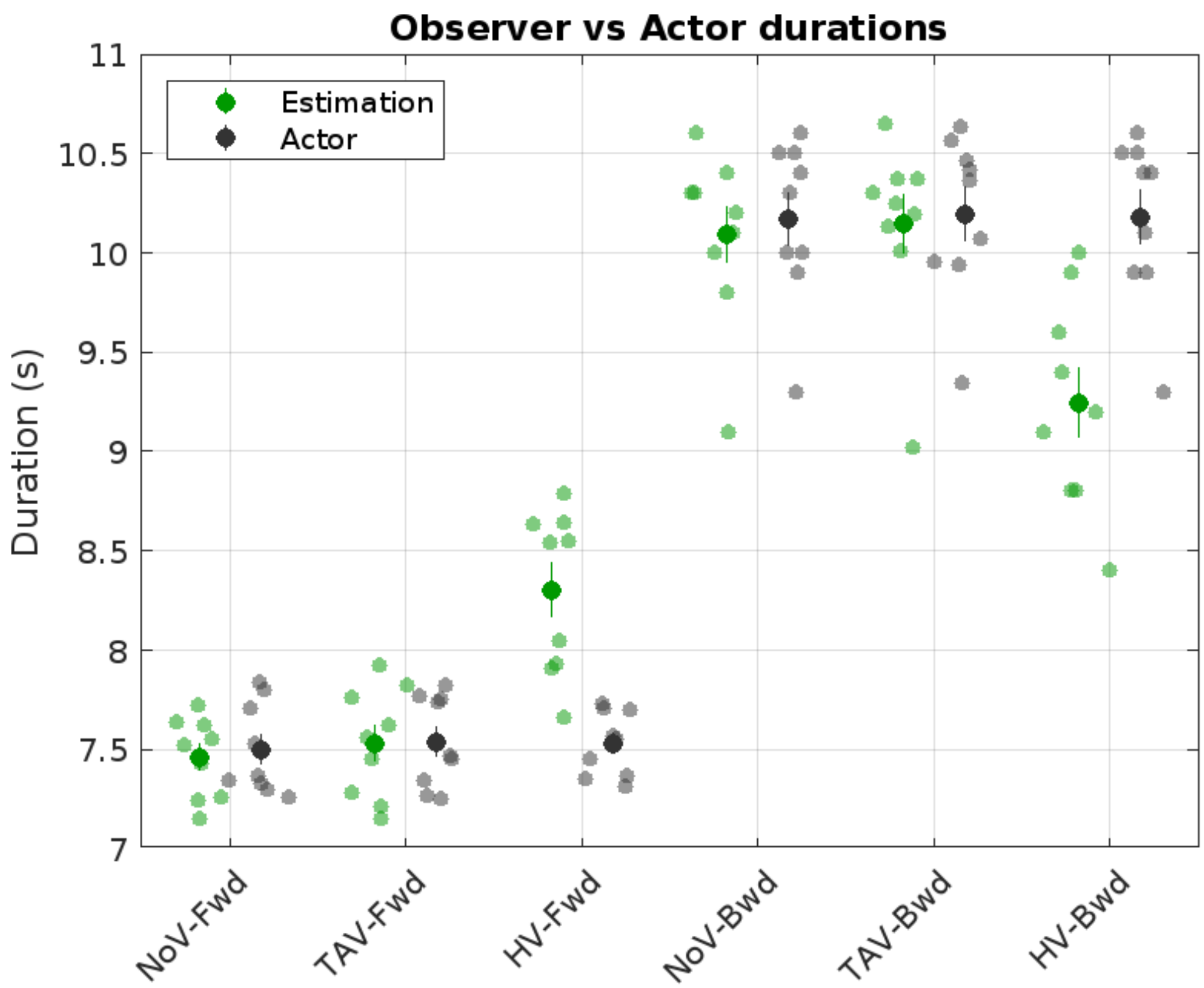

Figure S3: Observer estimations and actor locomotion durations across conditions, related to Experiment 3. Mean locomotion durations (± SEM) estimated by observers (green) and performed by actors (black) across six experimental conditions combining vibration type (NoV, TAV, HV) and movement direction (forward, backward). Dots represent individual participant values, illustrating the consistency of vibration effects across participants.

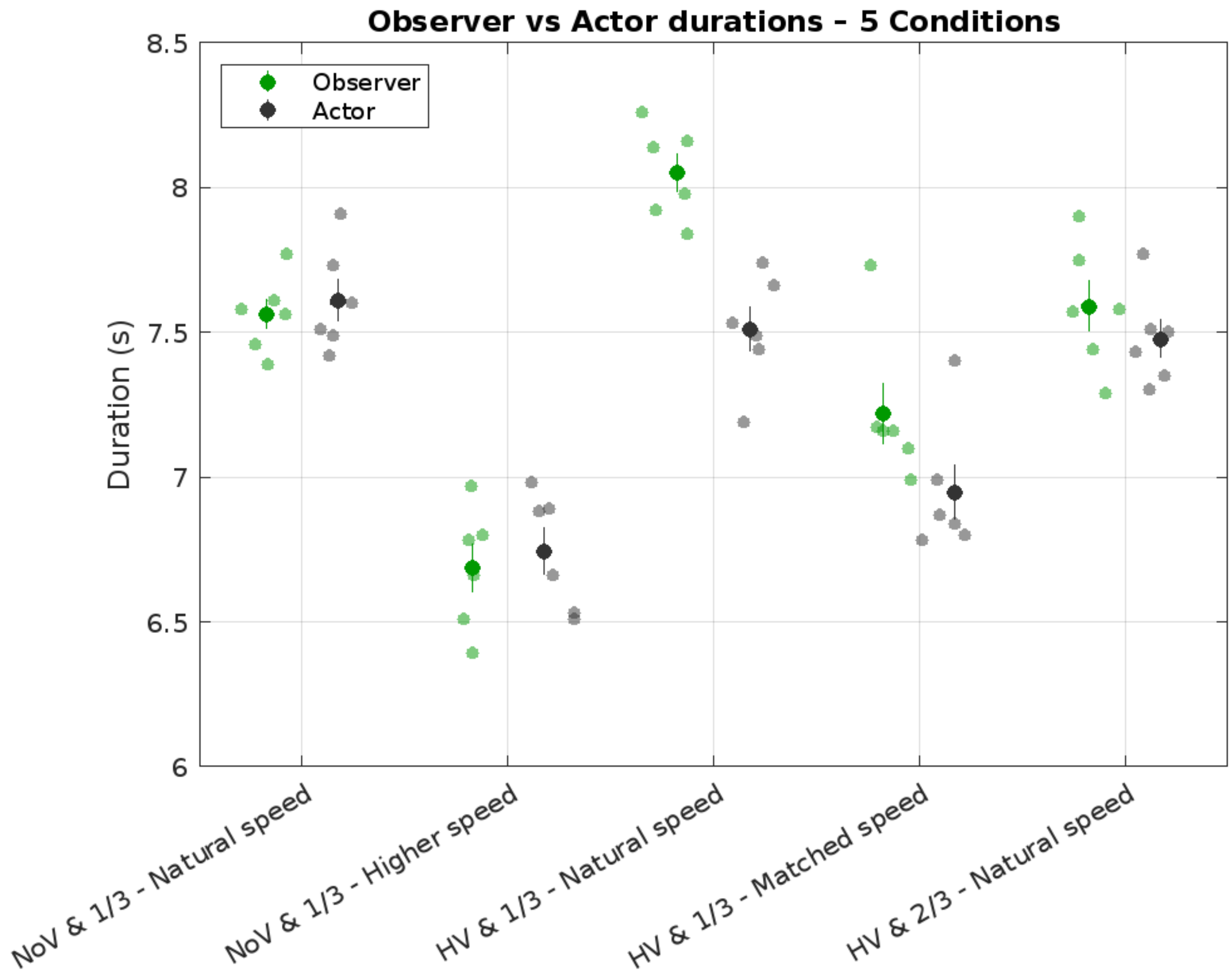

Figure S4: Locomotion inference under different visual and vibratory conditions, related to Experiment 4. Mean locomotion durations (± SEM) estimated by observers (green) and performed by actors (black) across five experimental conditions. Conditions differed in vibration (Vib vs NoV), amount of visual feedback (1/3 or 2/3 of the path visible), and walking speed (natural, higher, or matched). Dots represent individual participant values, illustrating the consistency of estimation patterns across participants.

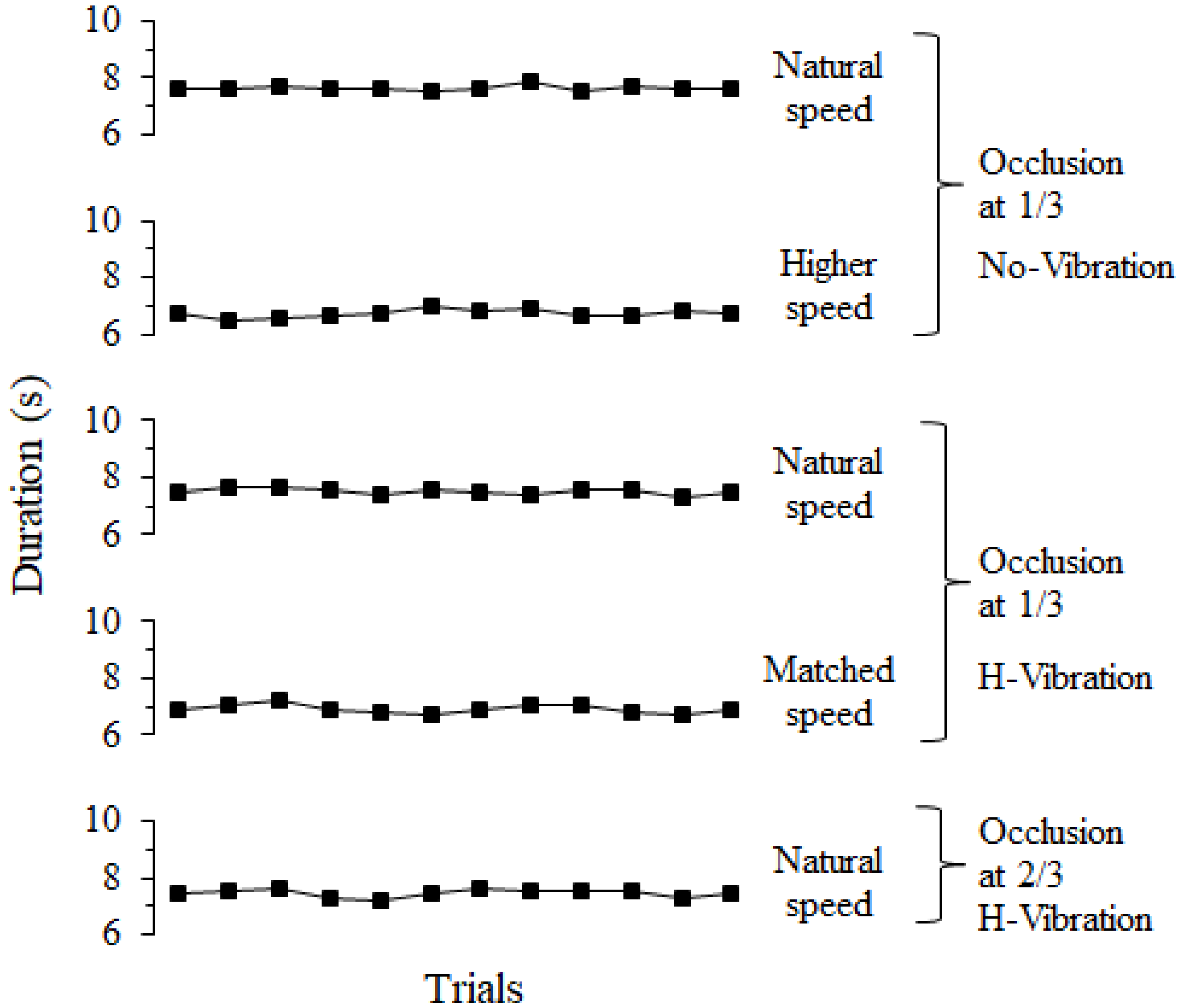

Figure S5: Trial-by-trial actor locomotion durations in Experiment 4, related to STAR methods Experiment 4. Mean trial-by-trial actor walking durations during forward locomotion across the five conditions of Experiment 4. Conditions 1–2 were performed without observer vibration, whereas conditions 3–5 involved hamstring vibration. Natural speed conditions corresponded to each observer's baseline walking speed; higher speed was set at +15%; matched speed corresponded to observer speed under vibration. Visual occlusion occurred after one-third of the movement (conditions 1–4) or two-thirds (condition 5). Trial stability analyses comparing trials 1–3 vs 10–12 showed no significant differences (all $t < 1$, $p > 0.1$).

Table S1: Mean locomotion durations in control experiments, related to Results – Experiments 1-3. Mean durations (± SD) of forward and backward locomotion measured during control experiments: Experiment 1a (imagined locomotion under 40 Hz vibration), Experiment 1b (imagined locomotion under different postural configurations), Experiment 2 (actual locomotion under 40 Hz vibration), and Experiment 3a (locomotion inference under 40 Hz vibration).

Duration (s)	Forward	Backward
Control experiment 1a		
No-vibration imagined locomotion	7.14 ±0.52	8.30 ±0.49
H-vibration imagined locomotion	7.12 ±0.49	8.32 ±0.52
Control Experiment 1b		
Erect Posture	7.01 ±0.55	
Posture corresponded to TA-vibration	7.06 ±0.63	
Posture corresponded to H-vibration	6.99 ±0.54	
Control experiment 2		
No-vibration actual locomotion	7.28 ±0.59	8.45 ±0.53
H-vibration actual locomotion	7.31 ±0.60	8.50 ±0.55
Control experiment 3a		
No-vibration actor	7.41 ±0.22	10.24 ±0.24
H-vibration observer (40Hz)	7.41 ±0.24	10.21 ±0.22

Table S2: Participant demographic characteristics, related to STAR Methods. Number of participants (N), sex distribution (female/male), and mean age (± SD) for each experiment and control condition included in the study. Experiments 1 and 2 involved the same participant cohort. Control experiments were performed with independent subsets or new participant groups as specified in the STAR Methods section. Race and ethnicity data were not collected.

Experiment / Control condition	N	Female	Male	Mean age (years ± SD)
Experiment 1 – Imagined locomotion	9	5	4	25.4 ± 2.7
Control Experiment 1a	5	2	3	24.4 ± 2.1
Control Experiment 1b	5	1	4	23.9 ± 1.8
Experiment 2 – Actual locomotion*	9	5	4	25.4 ± 2.7
Control Experiment 2	5	2	3	23.1 ± 3.0
Experiment 3 – Locomotion observation	9	4	5	25.2 ± 2.1
Control Experiment 3a	5	1	4	21.8 ± 2.9
Experiment 4 – Motor inference (visual feedback manipulation)	6	2	4	23.9 ± 2.2
